# Supplementary material for: Loss of the transcription factor MAFB limits β-cell derivation from human PSCs
Source: Nat Commun. 2020 Jun 2;11:2742. doi: 10.1038/s41467-020-16550-9 (PMC7265500; doi:10.1038/s41467-020-16550-9)
Supplement: Supplementary file 1 — Supplementary Information [file 41467_2020_16550_MOESM1_ESM.pdf]

**Supplementary Information**

# Loss of the transcription factor MAFB limits β-cell derivation from human PSCs

Russell et al.

# Supplementary Figure 1

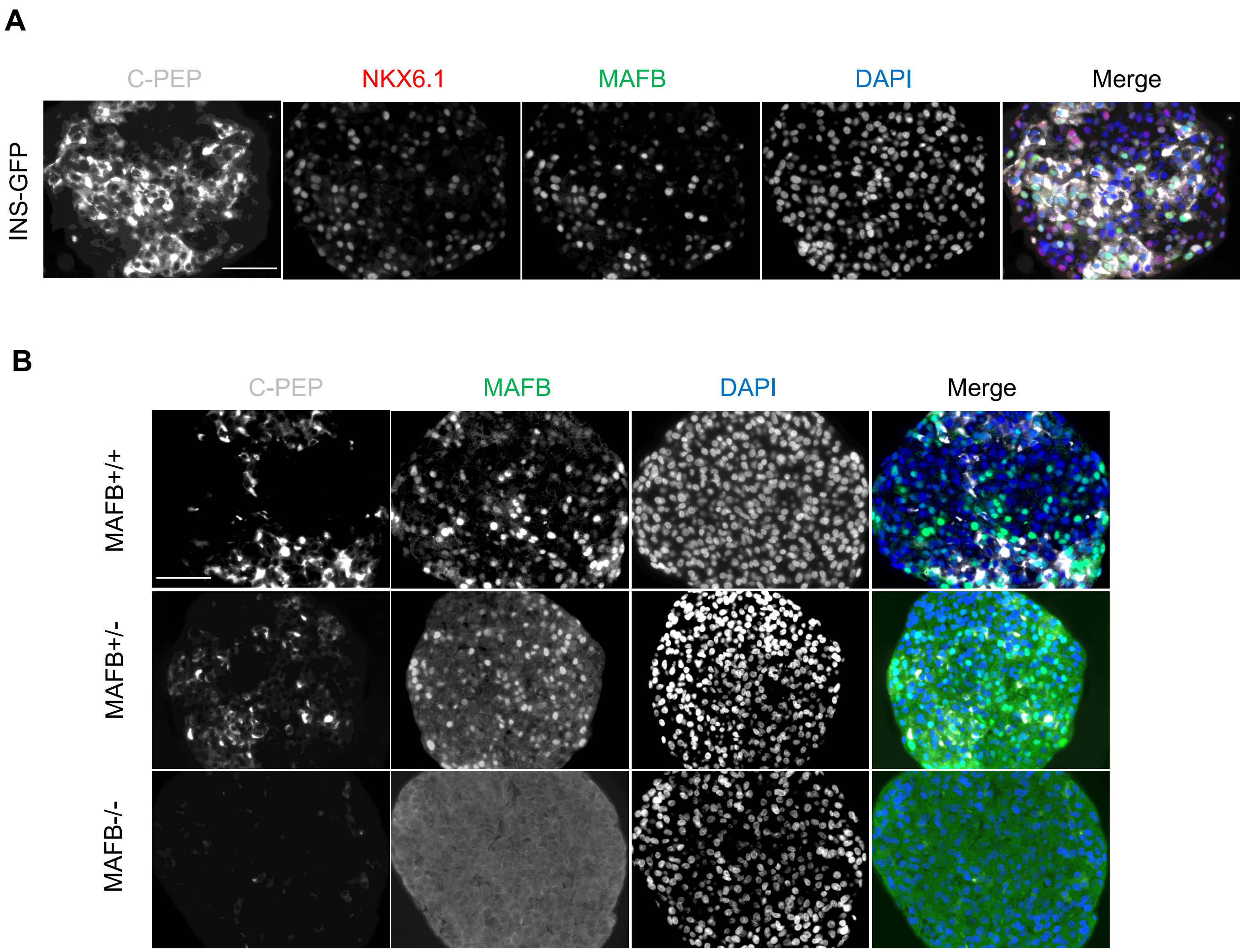


**Supplementary Figure 1. MAFB is expressed in hESC derived β-cells.**

(A) Representative IF images from 4 independent experiments of INS-GFP cells at the β-cell stage depicting C-PEP, NKX6.1, MAFB and DAPI. Scale bars, 50 μm.

(B) Representative IF images from 3 independent experiments from the β-cell stage of differentiation depicting C-PEP and MAFB expression in MAFB +/+, +/- and -/- cells. DAPI indicates nuclear staining. Scale bars, 50 μm.

# Supplementary Figure 2

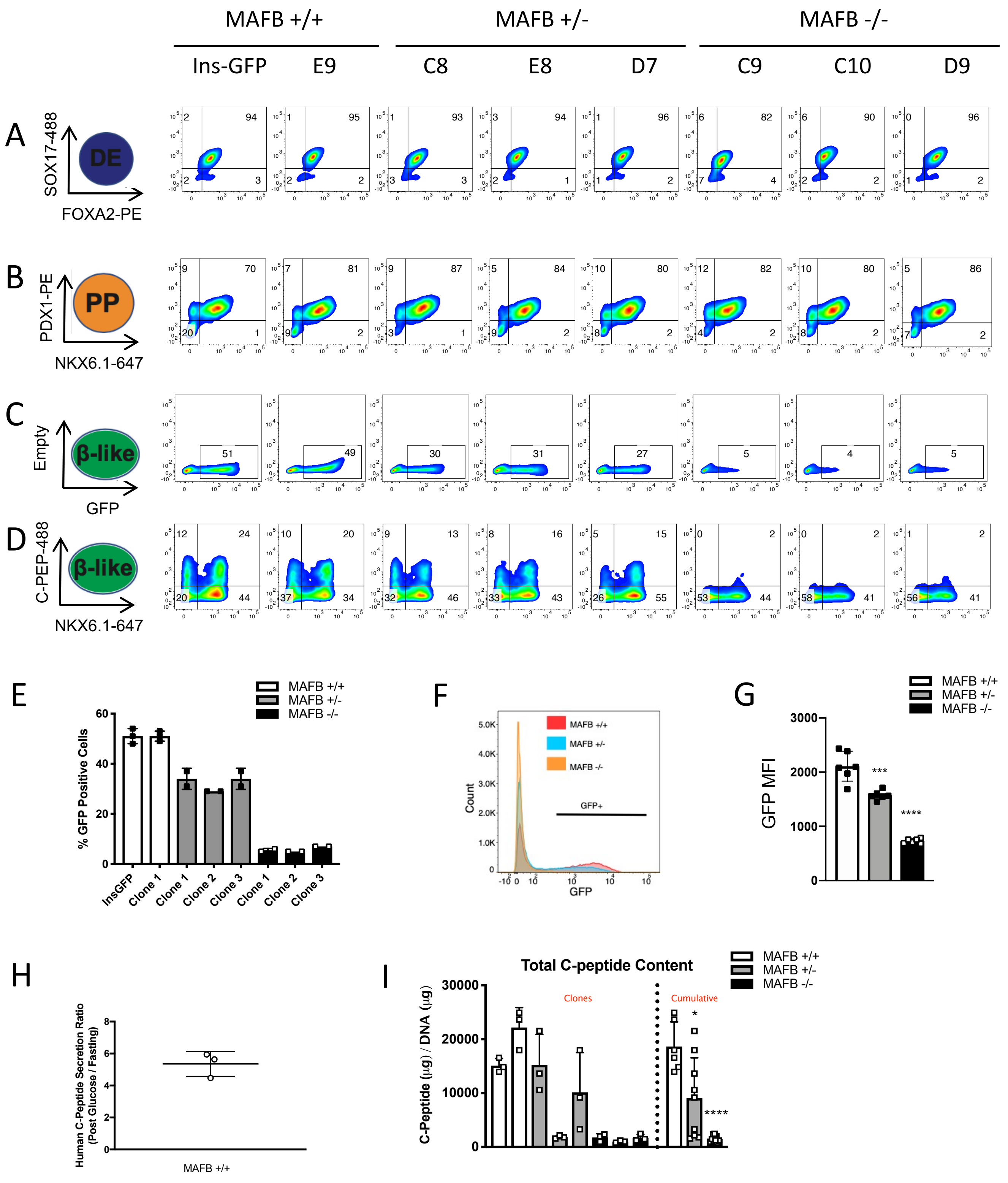

**Supplementary Figure 2. Loss of MAFB inhibits β-cell derivation.**

(A) Representative FC plots depicting the percentages of SOX17+ FOXA2+ at the DE stage for individual clones.

(B) Representative FC plots depicting the percentages of PDX1+ NKX6.1+ at the PP stage for individual clones.

(C) Representative FC plots depicting the percentages of GFP+ cells at the β-cell stage for individual clones.

(D) Representative FC plots depicting the percentages of C-PEP+ NKX6.1+ cells at the β-cell stage for individual clones.

(E) Quantification of GFP+ cells for individual clones the β-cell stage.

(F) Representative histograms and (G) Mean fluorescence intensity (MFI) of the GFP positive population in MAFB +/+, +/- and -/- cells. P values by one-way ANOVA followed by Dunnett's multiple comparisons test were ***P <0.0004 ****P < 0.0001. N = 6 independent experiments.

(H) Stimulation index of MAFB +/+ cells at 5 weeks post-transplantation. N = 3 independent mice.

(I) Total C-PEP content from individual clones at the β-like cell stage. N = 6 independent experiments.

P values by one-way ANOVA followed by Dunnett's multiple comparisons test were *P < 0.05, **P < 0.01, ***P <0.001, ****P < 0.0001. Data are presented as individual biological replicates and represent the mean ± SD.

Supplementary Figure 3

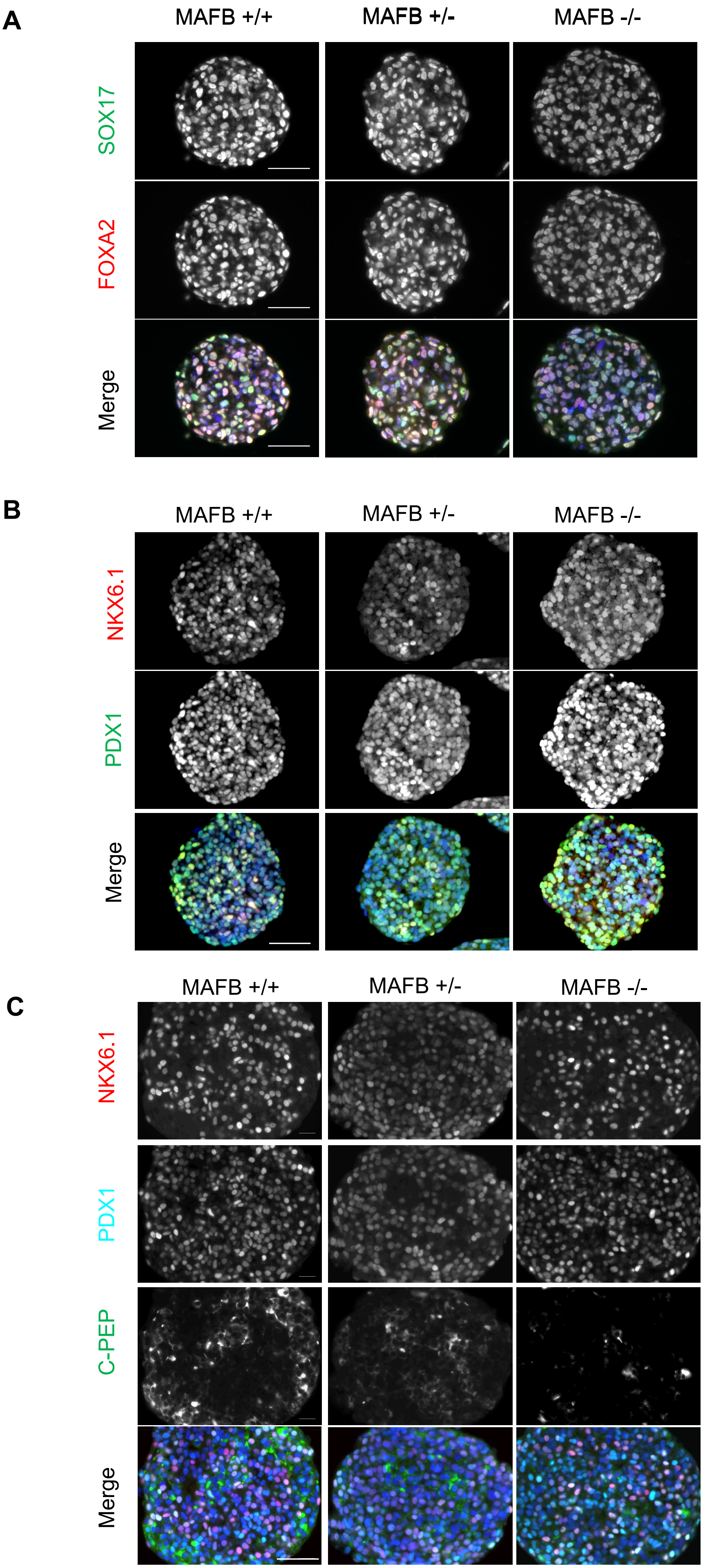

**Supplementary Figure 3. MAFB is important for β-cell derivation.**

(A) Representative images from 6 independent experiments at the DE stage of differentiation depicting SOX17 and FOXA2 staining. Scale bars, 50 μm.

(B) Representative images from 6 independent experiments at the PP stage of differentiation depicting PDX1 and NKX6.1 staining. Scale bars, 50 μm.

(C) Representative images from 6 independent experiments at the β-like cell stage of differentiation depicting C-PEP co-staining with PDX1 and NKX6.1. Scale bars, 50 μm.

# Supplementary Figure 4

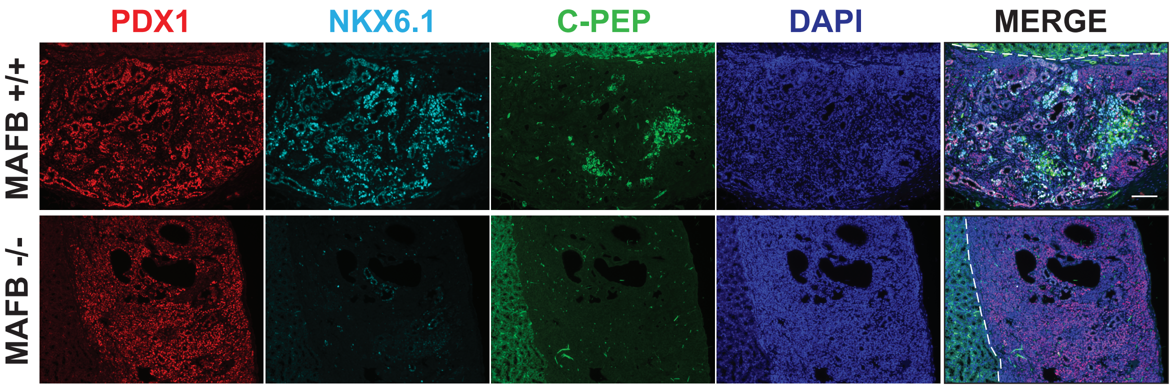


**Supplementary Figure 4. MAFB is required to generate functional β-cells *in vivo*.**

Representative IF images from 6 independent animals (3 MAFB+/+ and 3 MAFB-/-) for C-PEP co-staining with PDX1 and NKX6.1 of grafts removed from kidney capsules 8 weeks post-transplantation. The graft is delineated and marked from the kidney using dashed lines. Scale bars, 100 μm.

# Supplementary Figure 5

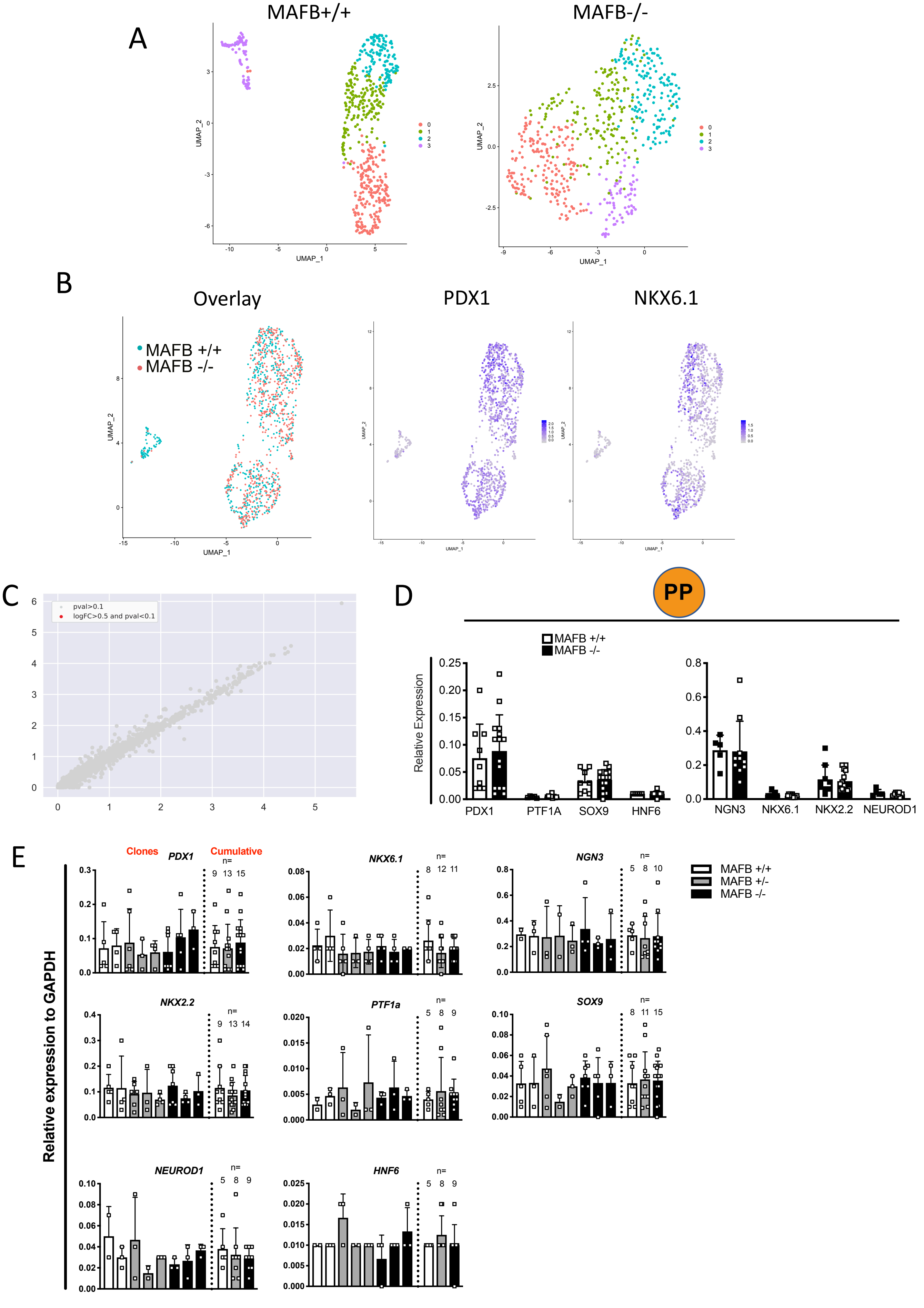

**Supplementary Figure 5. scRNA sequencing reveals MAFB-/- generate Pancreatic Progentior cells in equivalent manner to MAFB+/+ cells.**

(A) UMAP plot of MAFB +/+ and -/- cells at the PP stage of differentiation, color-coded by cell populations.

(B) UMAP projections of the integrated analysis of MAFB +/+ and -/- cells at the PP stage, color-coded by genotype (left) and normalized PDX1 and NKX6.1 expression (middle, right).

(C) Scatter plot showing the average gene expression (log scale) for MAFB +/+ and -/- cells at the PP cell stage. No genes are significantly differentially expressed (log fold change >0.5, adjusted p value < 0.1) between MAFB +/+ and -/- cells. P value calculated with MAST test.

(D) The relative mRNA expression patterns in MAFB +/+ and -/- cells of designated genes for pan-pancreatic (*PDX1, PTF1A, SOX9* and *HNF6*) or pro-endocrine (*NGN3, NKX6.1, NKX2.2* and *NEUROD1*) at the PP stage as measured by quantitative PCR analysis. Each data point represents an independent biological sample.

(E) The mRNA expression patterns of designated genes for individual clones for pancreatic (*PDX1, PTF1A, SOX9* and *HNF6*) or pro-endocrine genes (*NGN3, NKX6.1, NKX2.2* and *NEUROD1*) at the PP stage as measured by quantitative PCR analysis. Each data point represents an independent biological sample.

P values by unpaired two-tailed t-test were *P < 0.05, **P < 0.01, ***P < 0.001, ****P < 0.0001. Data are presented as individual biological replicates and represent the mean ± SD.

# Supplementary Figure 6

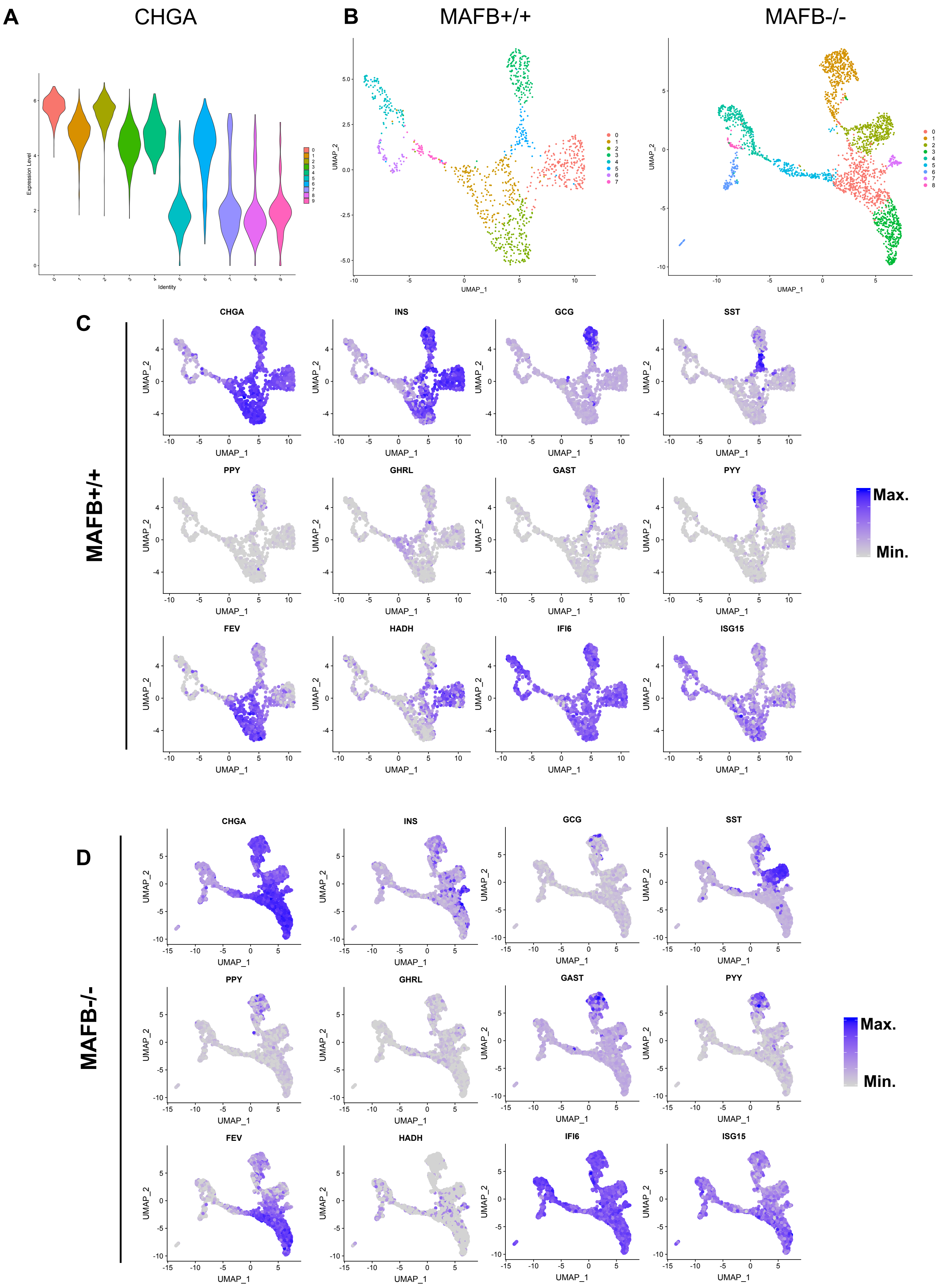

**Supplementary Figure 6. scRNA sequencing reveals MAFB regulates endocrine cell lineage commitment.**

(A) Violin plot of CHGA gene expression (normalized) in the merged MAFB +/+ and -/- cells from **Figure 4A**, color-coded by cell populations.

(B) UMAP plot of MAFB +/+ and -/- cells at the β-like cell stage of differentiation color-coded by cell identity.

(C,D) UMAP visualization of the log-transformed, normalized expression of selected genes at t he β-like cell stage of differentiation in (C) MAFB+/+ and (D) MAFB-/- cells.

# Supplementary Figure 7

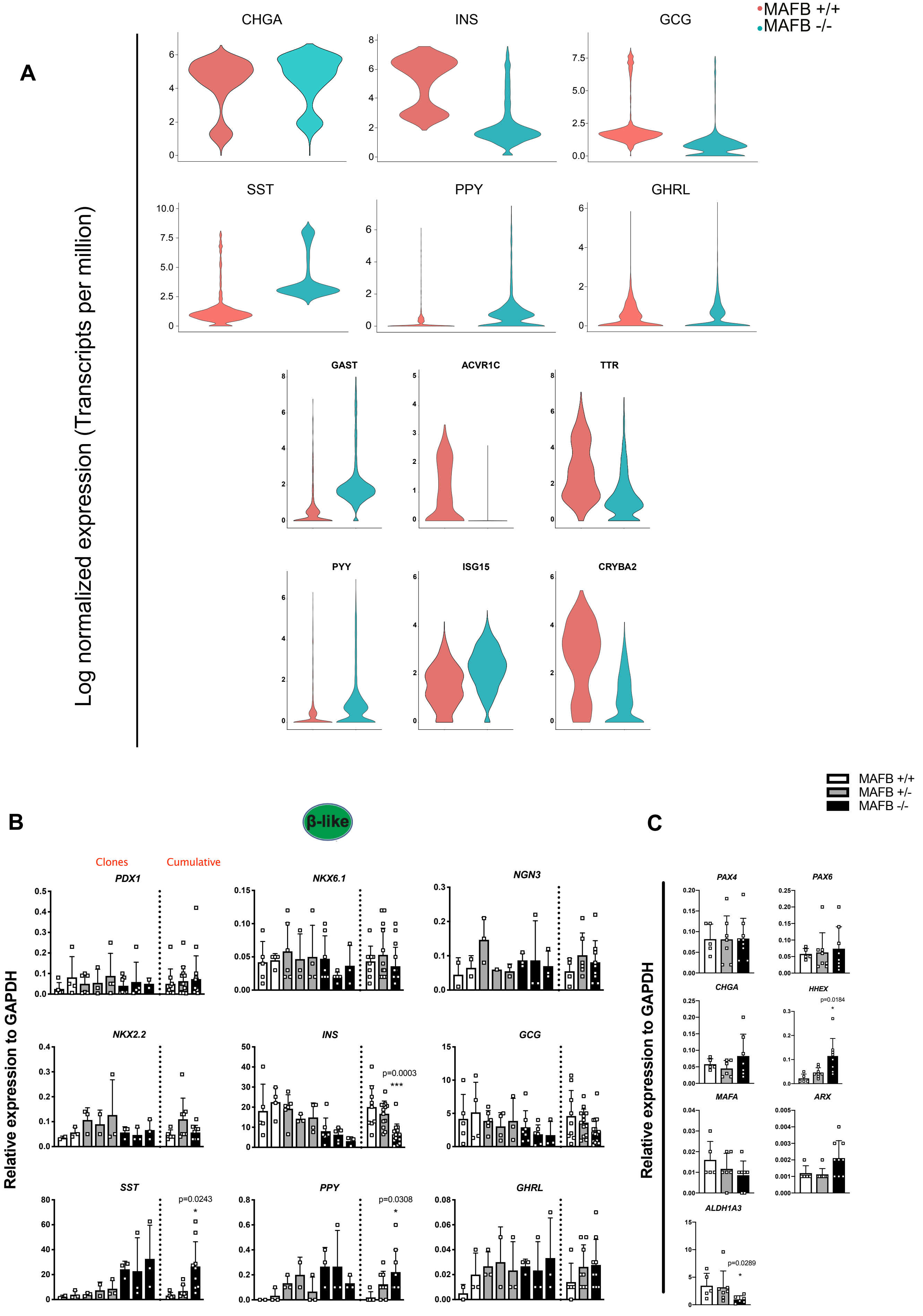

**Supplementary Figure 7. MAFB regulates endocrine hormone producing-cell lineage commitment.**

(A) Violin plot showing the expression of the top 5 genes and that are differentially expressed (Up and down regulated; fold change > 0.5, adjusted p value < 0.1) between MAFB +/+ and -/- cells in the β-like cell stage as well as the control endocrine genes *CHGA* and *GHRL*.

(B) The mRNA expression patterns of designated genes for individual clones for pro-endocrine (*NGN3*, *NKX6.1*, *NKX2.2* and *NEUROD1*) stages and islet hormones (*INS*, *GCG*, *SST*, *PPY* and *GHRL*) at the β-like cell stage as measured by quantitative PCR analysis. Each data point represents an independent biological sample.

(C) The mRNA expression patterns of genes implicated in endocrine cell fate (*PAX4*, *PAX6*, *HHEX*, *ARX*, *CHGA* and *MAFA*) as well as the β-cell de-differentiation marker *ALDH1A3* at the the β-like cell stage as measured by quantitative PCR analysis. Each data point represents an independent biological sample.

P values by unpaired two-tailed t-test were *P < 0.05, **P < 0.01, ***P < 0.001, ****P < 0.0001. Data are presented as individual biological replicates and represent the mean ± SD.

# Supplementary Figure 8

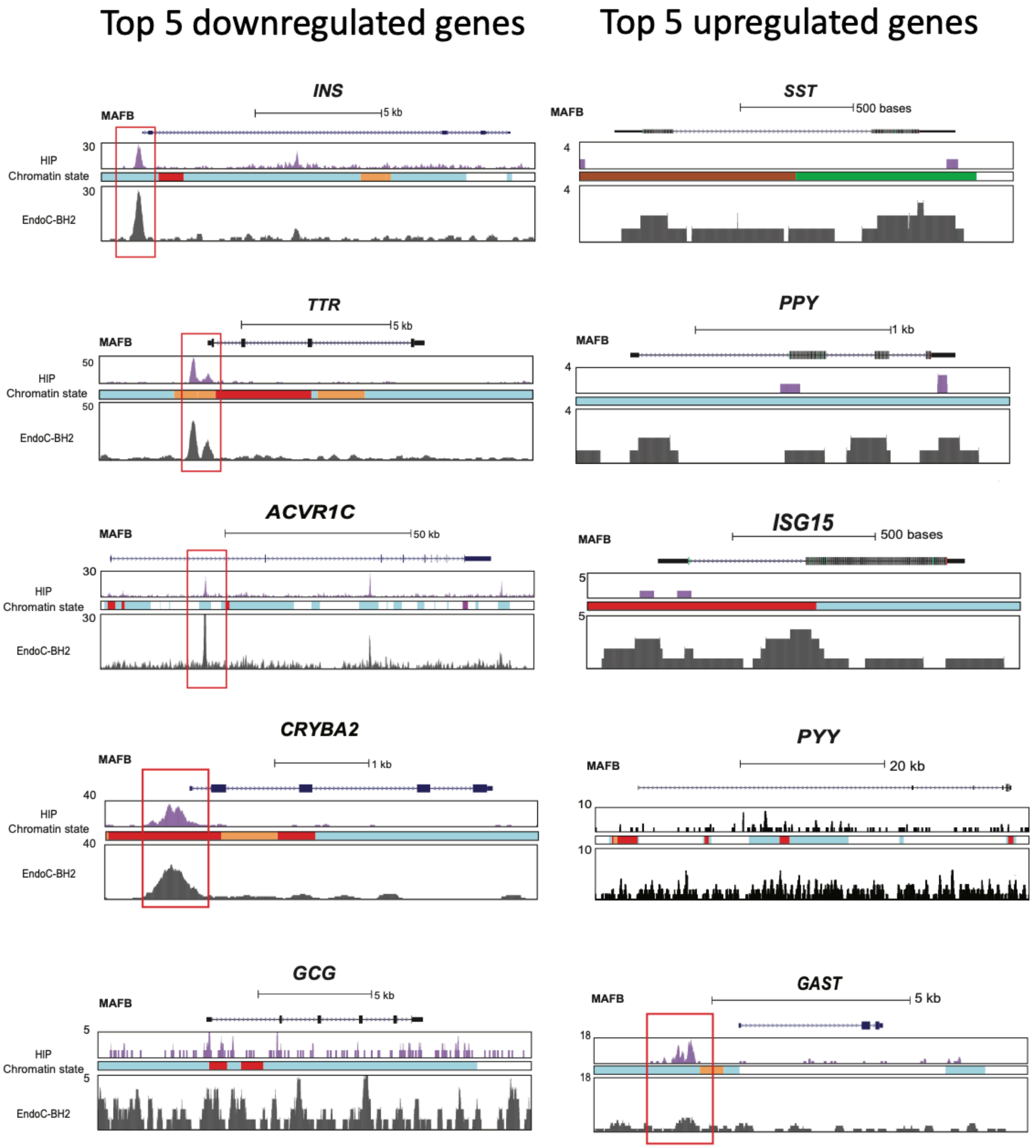


**Supplementary Figure 8. MAFB occupies α- and β-cell specific genes.**

ChIP-seq binding profiles for MAFB at the indicated *gene loci* in Human Islet Pancreas [1,2] and EndoC-BH2 cells. Tracks are set to the same scale for HIP and EndoC-BH2 cells per gene and the lengths of genes and direction of transcription are indicated by blue arrows. The top 5 up- and down-regulated genes identified in our MAFB +/+ and -/- β-cell differentiations in **Figure 4B** are outlined.

# Supplementary Figure 9

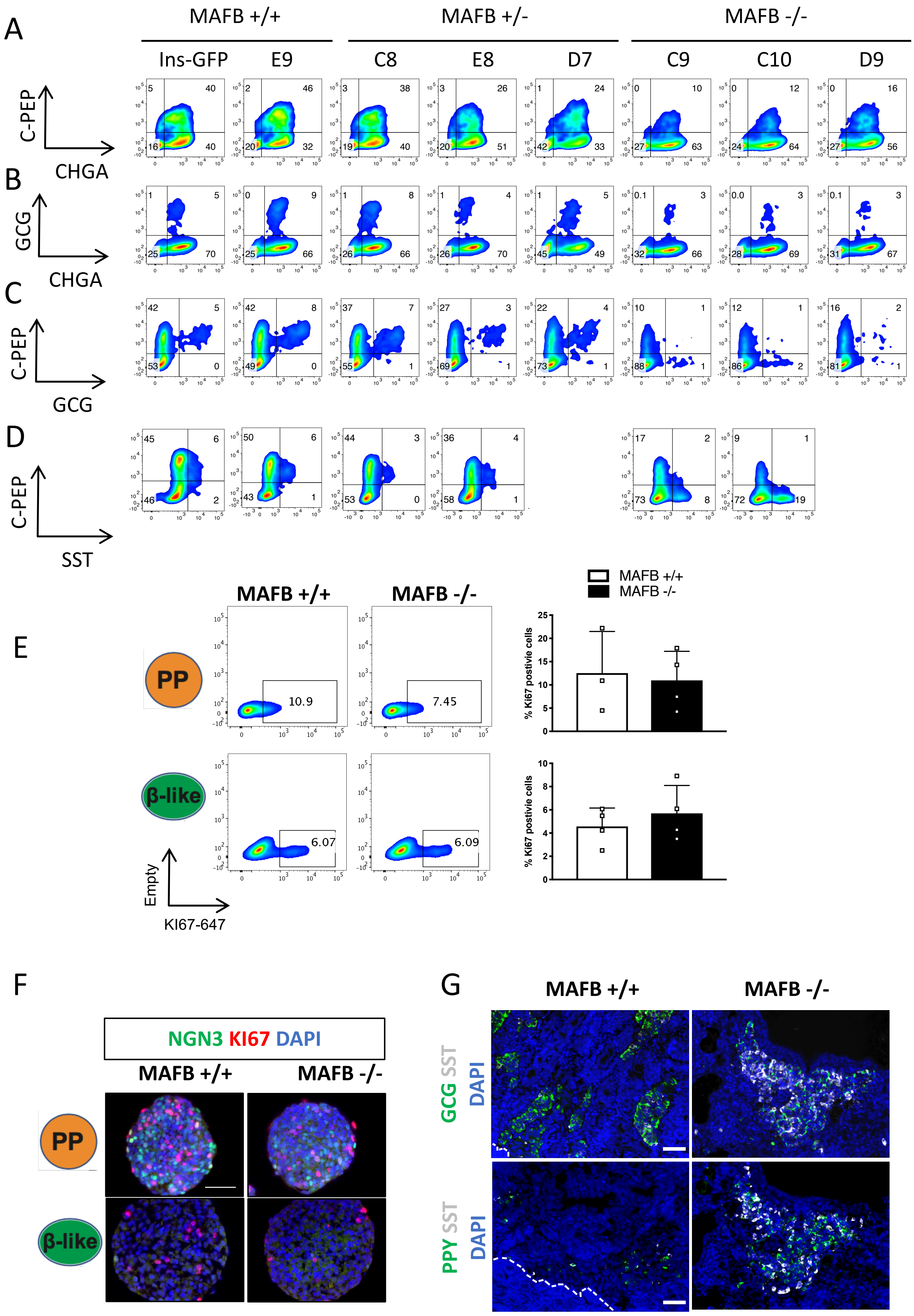

**Supplementary Figure 9. MAFB regulates differentiation of islet-hormone producing cell types.**

(A) Representative FC plots depicting the percentages of C-PEP+ CHGA+ cells for individual clones at the β-like cell stage.

(B) Representative FC plots depicting the percentages of CHGA+ GCG+ cells for individual clones at the β-like cell stage.

(C) Representative FC plots depicting the percentages of C-PEP+ GCG+ cells for individual clones at the β-like cell stage.

(D) Representative FC plots depicting the percentages of C-PEP+ SST+ cells for individual clones at the β-like cell stage.

(E) Representative FC plots depicting the percentages of KI67+ cells at the PP and β-cell stage and respective quantification (n=3).

(F) Representative IF images from 3 independent experiments of MAFB +/+ and -/- cells at the PP and β-cell stage depicting NGN3, KI-67 and DAPI. Scale bars, 50 μm.

(G) Representative IF images from 6 independent animals (3 MAFB+/+ and 3 MAFB-/-) for SST co-staining with GCG (upper panel) and PPY (lower panel) of grafts removed from kidney capsules 8 weeks post-transplantation of MAFB +/+ and -/- cells. The graft is delineated and marked from the kidney using dashed lines where indicated. Scale bars, 50 μm.

# Supplementary Figure 10

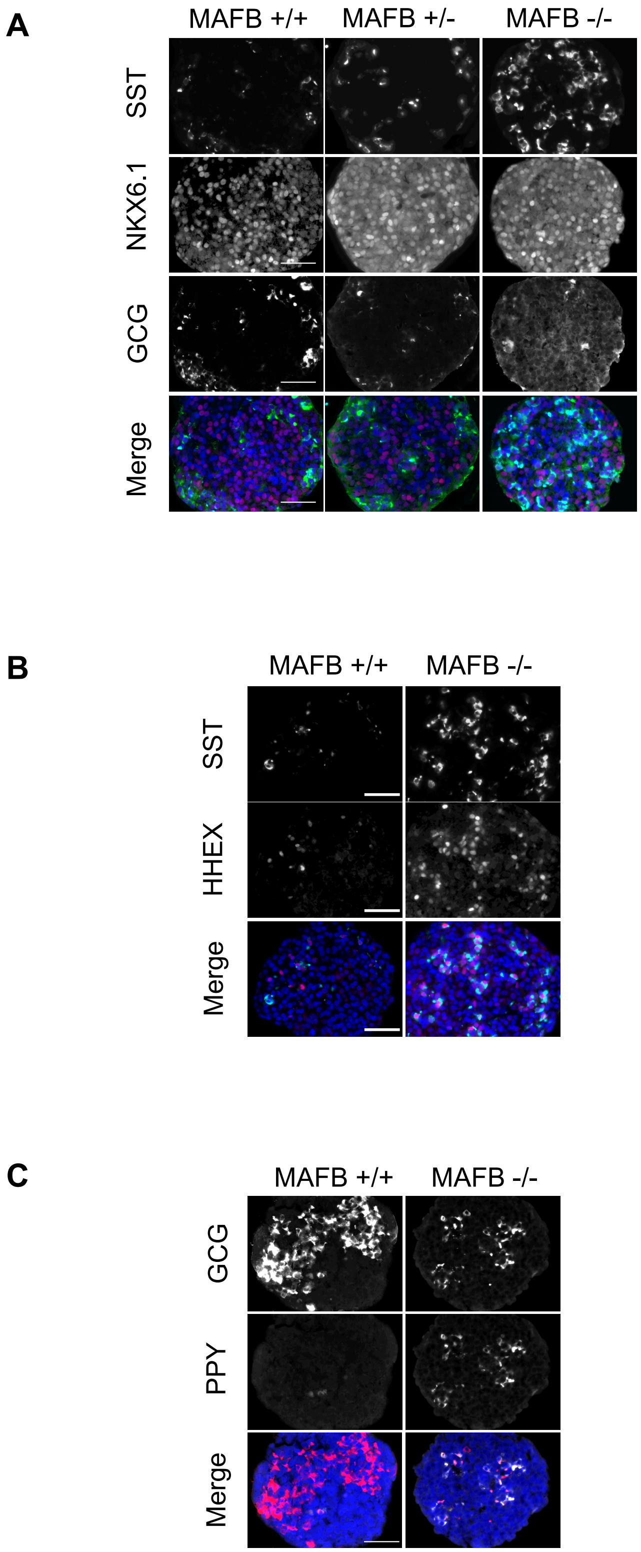

**Supplementary Figure 10. MAFB directs Pancreatic Endocrine cell lineage specification.**

(A) Representative IF images from 3 independent experiments the β-cell stage of differentiation depicting GCG, SST, NKX6.1 and DAPI. Scale bars, 50 μm.

(B) Representative IF images from 3 independent experiments the β-like cell stage of differentiation depicting SST and HHEX and DAPI. Scale bars, 50 μm.

(C) Representative IF images from 3 independent experiments the β-like cell stage of differentiation depicting PPY and GCG and DAPI. Scale bars, 50 μm.

# Supplementary Figure 11

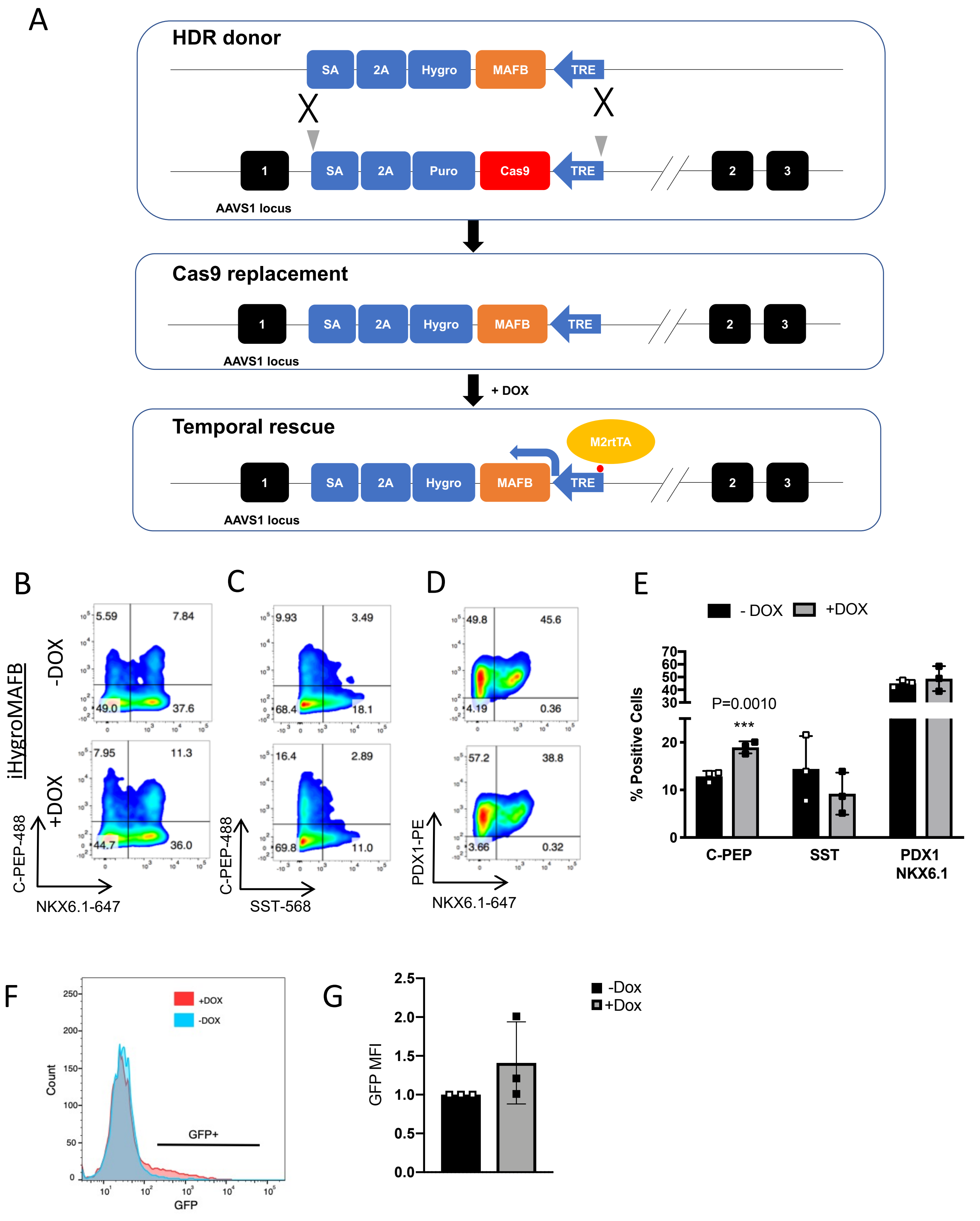

**Supplementary Figure 11. Rescue of MAFB in MAFB-/- cells.**

(A) Schematic outlining gene rescue and temporal control of *MAFB* expression in *MAFB-/-* hESCs.

(B) Representative FC plots depicting the percentages of C-PEP+ NKX6.1+ cells at the β-cell stage.
(C) Representative FC plots depicting the percentages of C-PEP+ SST+ cells at the β-cell stage.

(D) Representative FC plots depicting the percentages of PDX1+ NKX6.1+ cells at the β-cell stage.
(E) Quantification of data from B,C,D (n = 3).

(F) Representative histograms and (G) Mean fluorescence intensity (MFI) of the GFP positive population in -DOX and +DOX treated cells (n = 3).

P values by paired two-tailed t-test were *P < 0.05, **P < 0.01, ***P < 0.001, ****P < 0.0001. Data are presented as individual biological replicates and represent the mean ± SD.

# Supplementary Figure 12

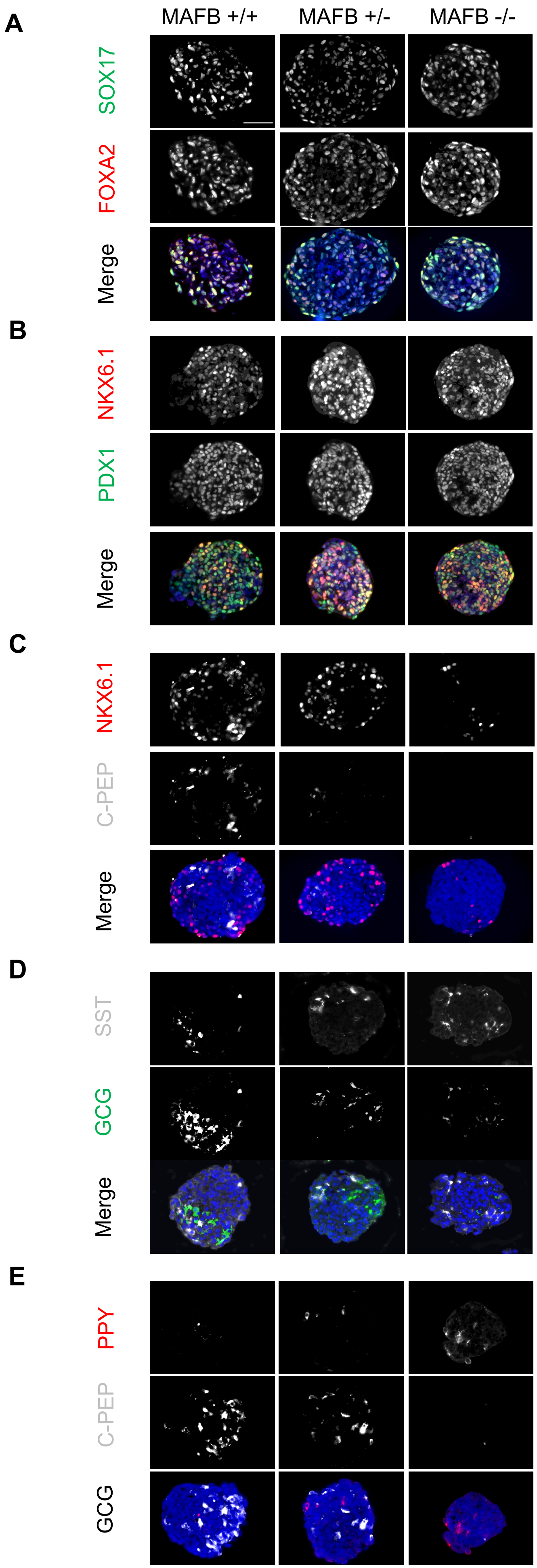

**Supplementary Figure 12. MAFB is critical to β-cell identity.**

(A – E) Representative IF images from 3 independent experiments at the DE, PP and β-like cell stages of differentiation for the respective markers as indicated. Scale bars, 50 µm.

# Supplementary Figure 13

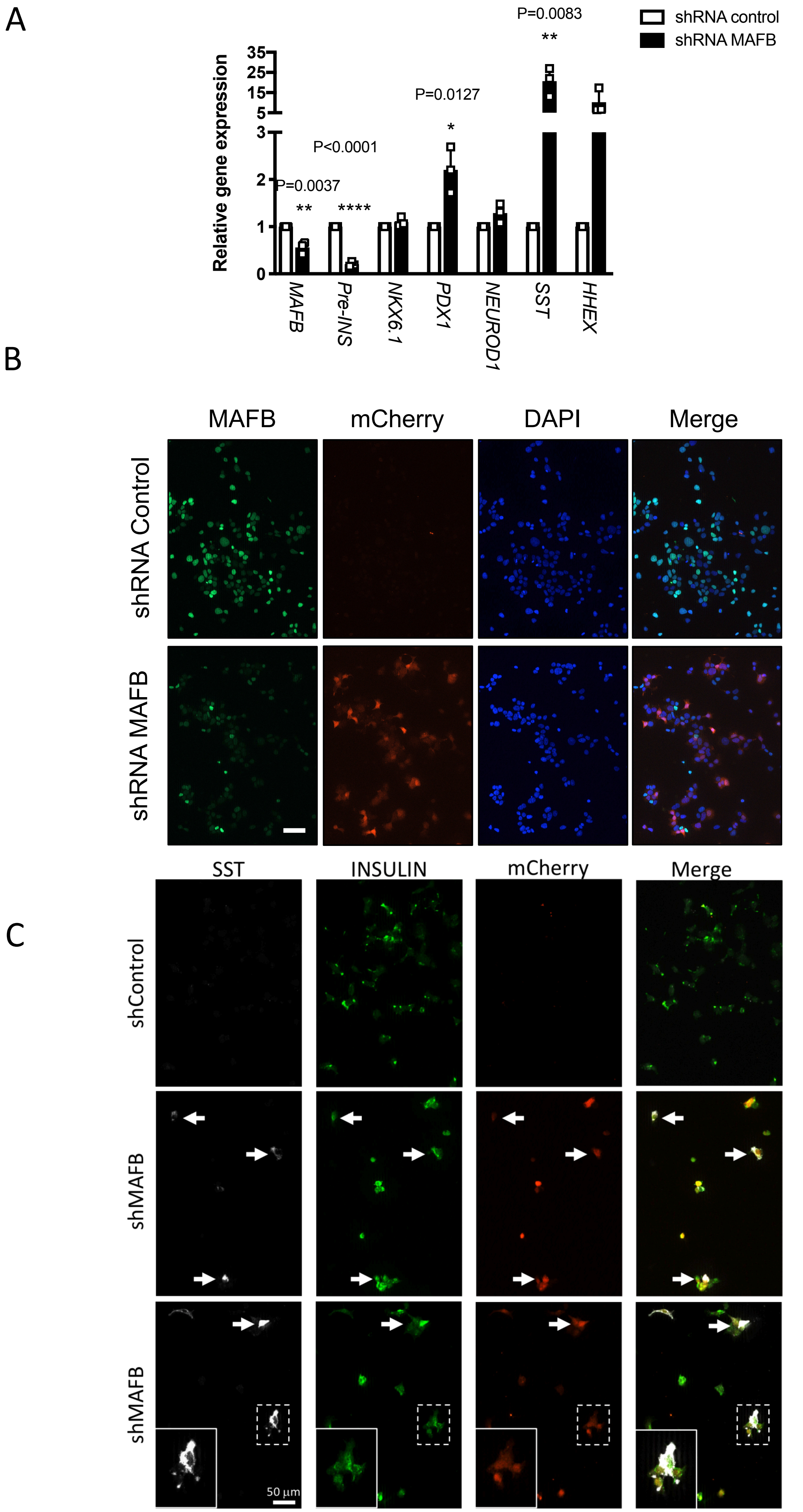

**Supplementary Figure 13. MAFB is important for β-cell identity in EndoC-βH2 cells.**

(A) The mRNA expression patterns of indicated genes for pancreatic and islet cell subtypes in EndoC-βH2 cells with shRNA control or MAFB knockdown as measured by quantitative PCR analysis (n=3).

(B) Representative IF images of EndoC-βH2 cells with shRNA control or MAFB depicting MAFB, mCherry and DAPI one week post lentiviral transduction. Scale bars, 50 μm.

(C) Representative IF images of EndoC-βH2 cells with shRNA control or MAFB depicting SST, INS, mCherry/GFP and DAPI one week post lentiviral transduction. Scale bars, 50 μm.

P values by paired two-tailed t-test were *P < 0.05, **P < 0.01, ***P < 0.001, ****P < 0.0001. Data are presented as individual biological replicates and represent the mean ± SD.

# Supplementary Figure 14

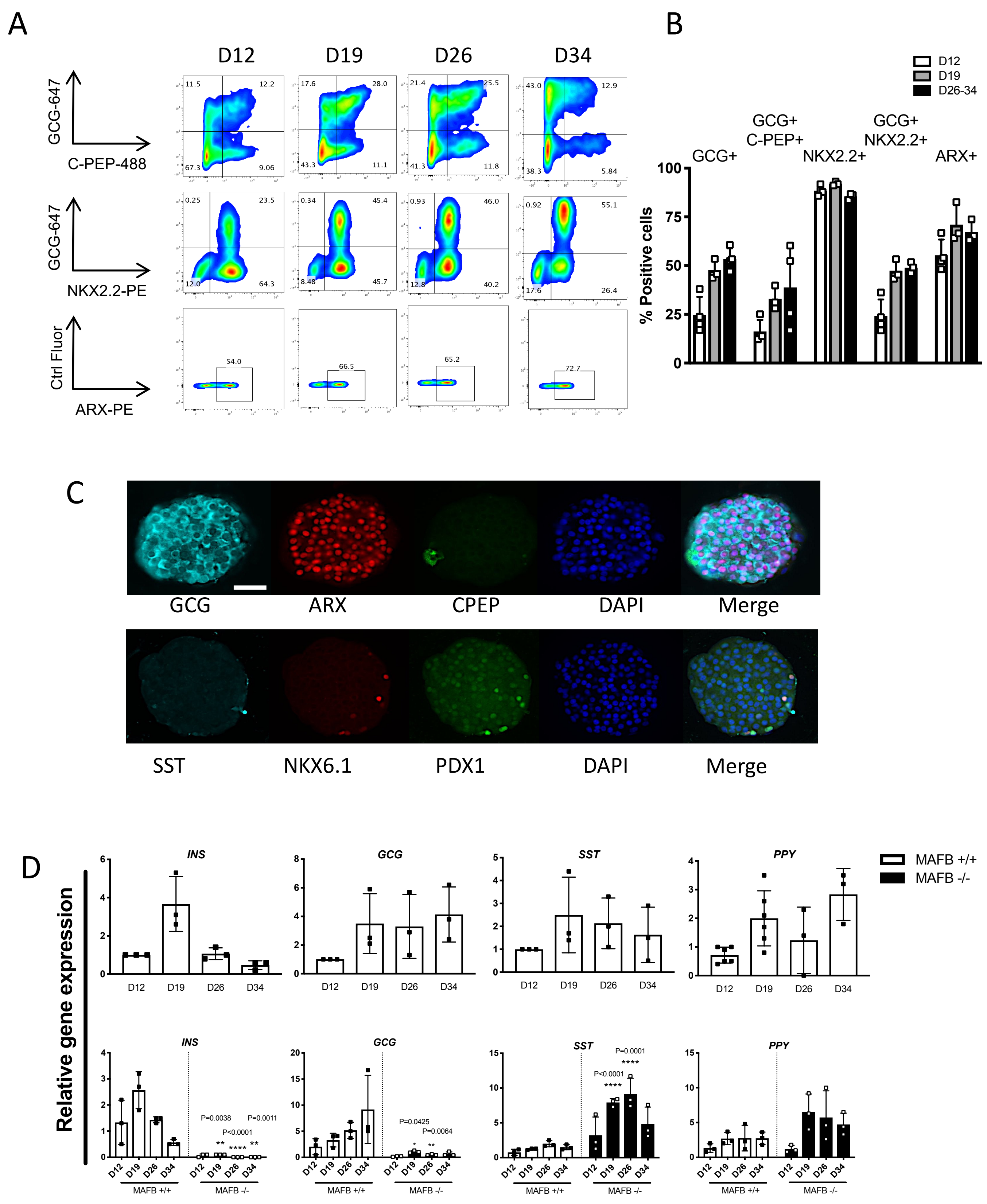

**Supplementary Figure 14. Characterization of human α-cell differentiation from INS-GFP hESCs.**

(A) Representative FC plots depicting the percentages of GCG+ C-PEP+ cells, GCG+ NKX2.2+ and ARX+ cell at the indicated stages of α-cell differentiation in INS-GFP cells.

(B) Quantification of FC data from A (n=3).

(C) Representative IF images from 3 independent experiments from differentiating α-cells depicting GCG, INS and ARX (top panel) and SST, NKX6.1 and PDX1 (lower panel) at D26. Scale bars, 50 μm.

(D) The mRNA expression patterns of islet hormones throughout α-cell differentiation at the indicated timepoints as measured by quantitative PCR analysis. Top panel – parental INS-GFP cell line. Lower panel – MAFB +/+ and -/- differentiations. (n=3).

P values by paired two-tailed t-test were *P < 0.05, **P < 0.01, ***P < 0.001, ****P < 0.0001. Data are presented as individual biological replicates and represent the mean ± SD.

# Supplementary Figure 15

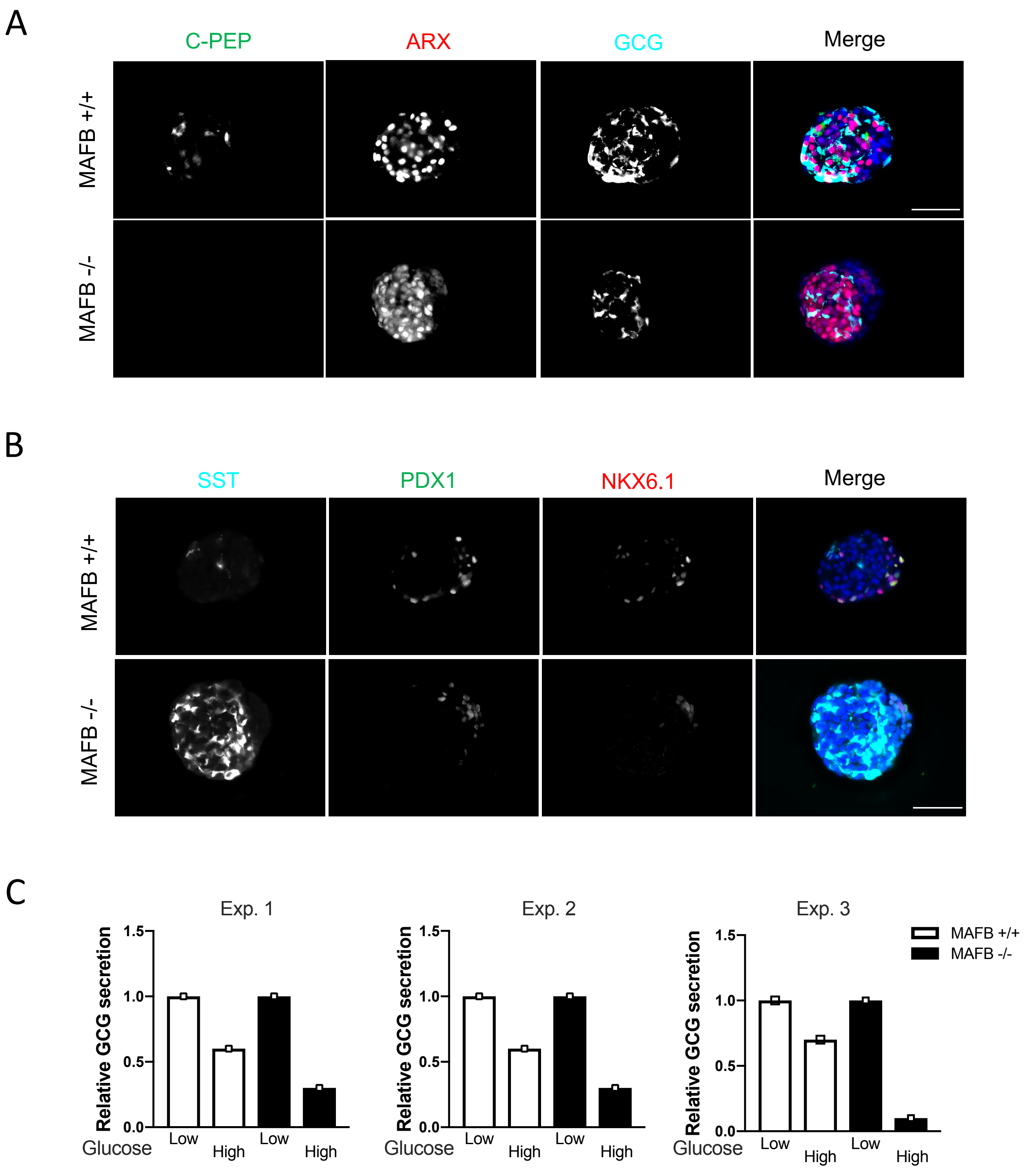


**Supplementary Figure 15. Loss of MAFB limits α-cell differentiation.**

(A,B) Representative IF images from 3 independent experiments at the α-cell stage depicting GCG, INS and ARX and PDX1, SST and NKX6.1 in d26 α-cells from MAFB +/+ and -/- differentiations. Scale bars, 50 µm.

(C) Quantification of GCG secretion in response to low and high glucose from MAFB +/+ and -/- cells at d26-34 *in vitro*. 3 independent experiments are shown.

# Supplementary Figure 16

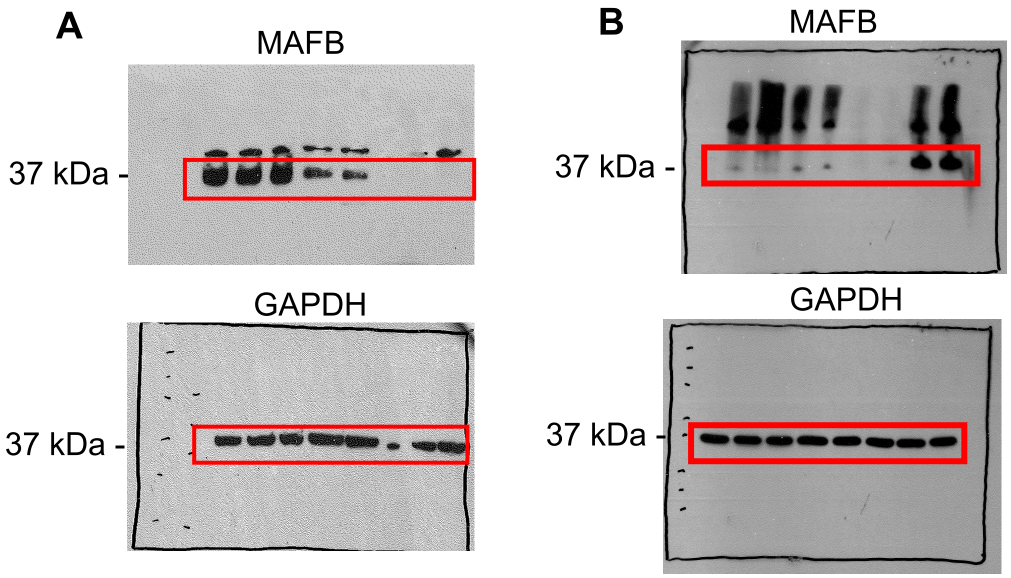


**Uncropped immunoblots from Figure 1**

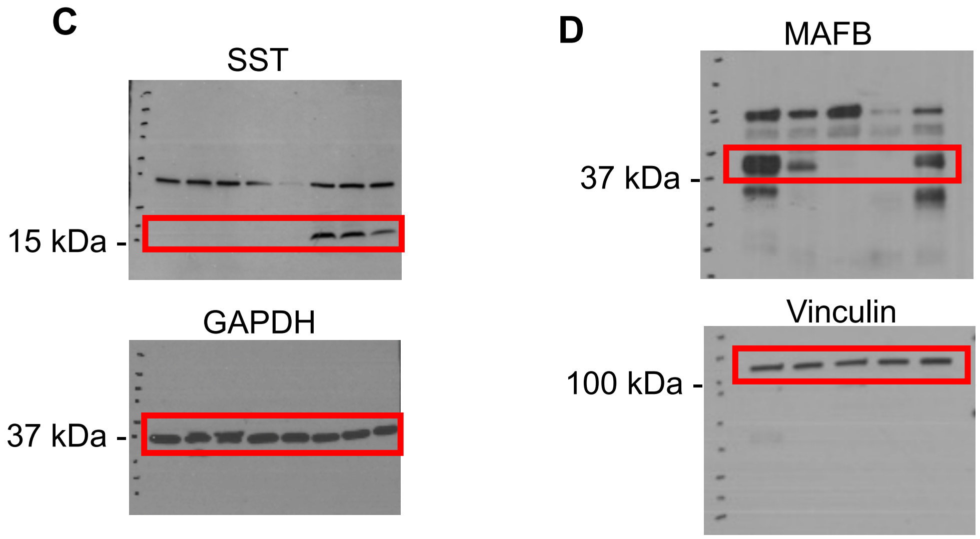


**Uncropped immunoblots from Figure 4**

**Uncropped immunoblots from Figure 5**

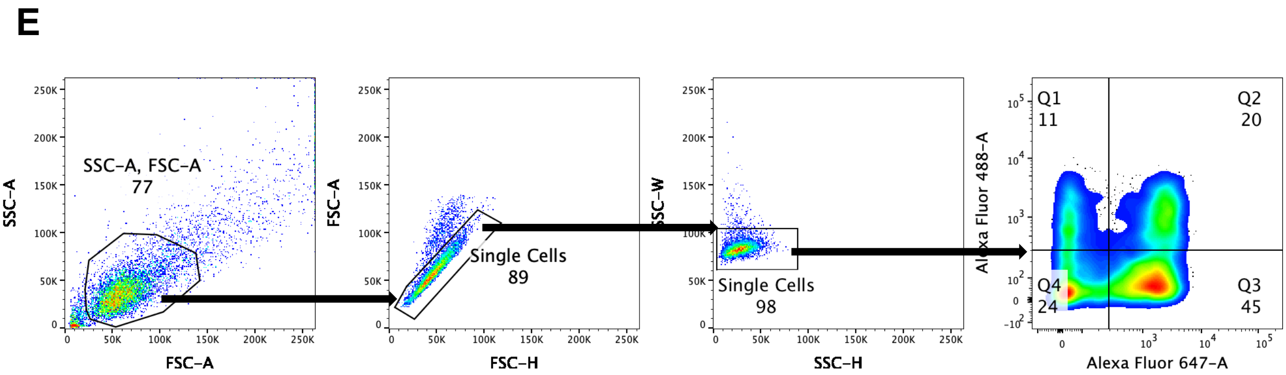


**Gating Strategy for Flow Cytometry Analysis**

**Supplementary Figure 16.**

(A-D) Uncropped western blots.

(E) Flow Cytometry gating strategy.

**Supplementary References:**

1 Pasquali, L. *et al.* Pancreatic islet enhancer clusters enriched in type 2 diabetes risk-associated variants. *Nat Genet* **46**, 136-143, doi:10.1038/ng.2870 (2014).

2 Mularoni, L., Ramos-Rodriguez, M. & Pasquali, L. The Pancreatic Islet Regulome Browser. *Front Genet* **8**, 13, doi:10.3389/fgene.2017.00013 (2017).

**Supplementary Table 1. hESC differentiation into β-like cells – Backbone Media Formulation**

| Day | Media components | | | | |
|---|---|---|---|---|---|
| D0 | DMEM/F-12 | 1xGlutamax | 1xMEM-NEAA | 1x 2-Mercaptoethanol | 4% KSR |
| D1-5 | RPMI | 1xGlutamax | | | |
| D6-7 | DMEM | 1xGlutamax | | | |
| D8 | DMEM | 1xGlutamax | | | |
| D9-11 | DMEM | 1xGlutamax | | | |
| D12-20 | DMEM | 1xGlutamax | | | |

**Supplementary Table 2. hESC differentiation into β-like cells – Media components**

| Day | Media | Supplement |
|---|---|---|
| D0 | Seed hESCs 5.5 million cells/ well in 6 well plates with D0 media | |
| | Day0 | HERβ1 10ng/ml; Activin A 10ng/ml; FGF-2 4.16ng/ml |
| D1 | Rinse cells with DPBS (w/o $Ca^{2+}$, $Mg^{2+}$) for 5 min and added Day 1 differentiation media | |
| | Day 1 | FBS 0.2%; Activin A 100ng/ml; ITS 0.02X; Wnt-3a 50ng/ml |
| D2 | Day 2 | FBS 0.2%; Activin A 100ng/ml; ITS 0.05X |
| D3 | Day 3 | FBS 0.2%; KGF 25ng/ml; ITS 0.1X; TGF-β IV 2.5μM |
| D4-5 | Day 4-5 | FBS 0.2%; KGF 25ng/ml; ITS 0.1X |
| D6-7 | Day 6-7 | B27 1X; TTNPB 3nM |
| D8 | Day 8 | B27 1X; TTNPB 3nM; EGF 50ng/ml |
| D9-11 | Day 9-11 | B27 1X; KGF 50ng/ml; EGF 50ng/ml |
| D12-20 | Day 12-20 | B27 1X; N-acetly L-cysteine 1mM; NEAA 1X; Heparin 10ug/ml; Zinc sulfate 10μM; Alk5i II 10uM; T3 136ng/mL; LDN 500nM; XXi 2μM; Vitamin C 500μM |

**Supplementary Table 3. CRISPR gRNA target sequences for targeting MAFB.**

| Gene + guide no | gRNA Target sequence | PAM |
|---|---|---|
| MAFB gRNA_1 | GGTGTGTCTTCTGTTCGGTC | GGG |
| MAFB gRNA_2 | TCTTCACGTCGAACTTGCGC | AGG |
| MAFB gRNA_3 | ACACGGAGTGCTGAGCGGTG | TGG |

**Supplementary Table 4. PCR and sequencing primers used for genotyping and sequencing**

| Primer | Sequence (5'to 3') |
|---|---|
| MAFB Seq Forward | GTGGAGAGGCAAGCGGAG |
| MAFB Seq Reverse | GGGTTCATCTGCTGGTAGT |

**Supplementary Table 5. Clonal Cell line sequence information.**

| Clone Name INS-GFP cells | Allele 1 | Allele 2 | Mutation | MAFB |
|---|---|---|---|---|
| E9 | TACACGGAGTGCTGAGCGGTGTGG | TACACGGAGTGCTGAGCGGTGTGG | WT / WT | +/+ |
| C8 | TACACGGAGTGCTGAGCGGTGTGG | TACACGGAGTGCTGAGC TACACGGAGTGTGGAGCTACACGGAGCTACACGGA -GTGTGG | WT / 35bp insertion, 1bp deletion | +/- |
| D7 | TACACGGAGTGCTGAGCGGTGTGG | TACACGGAGTGCTGAG ----------- | WT / 11bp deletion | +/- |
| E8 | TACACGGAGTGCTGAGCGGTGTGG | TACACGGAGTGCTGAGCGG GTGTGG | WT / 1bp insertion | +/- |
| C9 | TACACGGAGTGCTGAG ----------- | TACACGGAGTGCTGAGCGG GTGTGG | 11 bp deletion / 1bp insertion | -/- |
| C10 | TACACGGAGTGCTGAG ---------- | TACACGGAGTG---------- TGTGG | 10 bp deletion / 10 bp deletion | -/- |
| D9 | TACACGGAGTGCTGAGCGG GTGTGG | TACACGGAGTGCTGAGCGG GTGTGG | 1 bp insertion / 1 bp insertion | -/- |

| iPSCs | Mutation | MAFB |
|---|---|---|
| WT | WT / WT | +/+ |
| Het | WT / 1bp insertion | +/- |
| KO | -83bp / -83bp | -/- |

**Supplementary Table 6. Differentially Expressed genes from MAFB +/+ and -/- cells at the beta-like stage (P_adj<0.1, FC>0.5)**

| Upregulated | Gene | avg_logFC | p_val_adj |
|---|---|---|---|
| 1 | GAST | 2.316356715 | 5.47E-303 |
| 2 | SST | 2.041943535 | 3.41e-312 |
| 3 | PPY | 1.413802582 | 2.66E-86 |
| 4 | PYY | 0.855968146 | 5.21E-40 |
| 5 | ISG15 | 0.708019373 | 2.08E-106 |
| 6 | LY6E | 0.70691153 | 1.69E-110 |
| 7 | CGA | 0.694610481 | 5.11E-26 |
| 8 | TPPP3 | 0.687042188 | 2.92E-55 |
| 9 | RGS4 | 0.677778669 | 6.87E-62 |
| 10 | HSPA1B | 0.669118669 | 6.82E-128 |
| 11 | KCTD12 | 0.627579284 | 4.84E-73 |
| 12 | FXYD2 | 0.600230106 | 2.50E-15 |
| 13 | APOC1 | 0.561225169 | 1.98E-50 |
| 14 | CTSB | 0.554216445 | 4.72E-66 |
| 15 | HSPA1A | 0.549208538 | 2.61E-62 |
| 16 | TIMP3 | 0.53469464 | 5.92E-48 |

| Downregulated | Gene | avg_logFC | p_val_adj |
|---|---|---|---|
| 1 | GCG | -2.014125141 | 2.97E-196 |
| 2 | INS | -1.934440177 | 0 |
| 3 | CRYBA2 | -1.931697312 | 7.71E-270 |
| 4 | TTR | -1.227343372 | 4.19E-141 |
| 5 | ACVR1C | -1.202830614 | 8.46E-202 |
| 6 | SLC30A8 | -0.964682775 | 3.13E-147 |
| 7 | PPP1R1A | -0.829768652 | 1.25E-177 |
| 8 | MT1G | -0.789324756 | 5.68E-20 |
| 9 | PCP4 | -0.767236561 | 4.91E-23 |
| 10 | STMN2 | -0.766230297 | 6.22E-121 |
| 11 | NPTX2 | -0.688057809 | 6.09E-95 |
| 12 | COL5A2 | -0.680313646 | 5.13E-49 |
| 13 | MT2A | -0.655040748 | 2.85E-09 |
| 14 | HMGB3 | -0.641933516 | 2.24E-137 |
| 15 | EPHX1 | -0.638270828 | 1.44E-161 |
| 16 | HADH | -0.638030748 | 8.31E-132 |
| 17 | MT1X | -0.601419028 | 3.06E-25 |
| 18 | ERO1LB | -0.596307901 | 7.24E-46 |
| 19 | NEUROD1 | -0.574711197 | 3.39E-93 |
| 20 | CRISPLD1 | -0.546023471 | 1.81E-74 |
| 21 | NKX6-1 | -0.54402969 | 8.58E-86 |
| 22 | GAL | -0.537813148 | 2.19E-28 |
| 23 | AIF1 | -0.536504473 | 1.62E-127 |
| 24 | SLC7A8 | -0.536149322 | 1.43E-122 |
| 25 | SNORA76 | -0.509134644 | 8.98E-136 |
| 26 | RP11-788H18.1 | -0.504352542 | 5.51E-105 |

Supplementary Table 7. Differentially Expressed genes from MAFB +/+ and -/- cells in INS NKX6.1 high (Beta-like) cells at D20
(P_adj<0.1, FC>0.5)

| | Gene | avg_logFC | p_val_adj |
|---|---|---|---|
| **Upregulated** | SST | 1.7366719 | 1.96E-60 |
| | GAST | 1.66897755 | 3.46E-35 |
| | TDO2 | 1.20887123 | 4.11E-14 |
| | GIP | 1.13050186 | 0.00014082 |
| | DLK1 | 1.11721996 | 3.94E-13 |
| | CALB2 | 1.04430784 | 3.19E-19 |
| | LY6E | 1.03407021 | 2.42E-23 |
| | DDC | 1.01089582 | 1.35E-25 |
| | KCTD12 | 0.99970662 | 4.50E-23 |
| | RGS4 | 0.9782056 | 1.94E-13 |
| | WLS | 0.9635128 | 6.22E-30 |
| | APOB | 0.94060072 | 2.25E-10 |
| | PYY | 0.90482411 | 5.19E-08 |
| | RGS2 | 0.87481822 | 1.48E-18 |
| | HSPA1B | 0.85069924 | 2.87E-22 |
| | IGFBP3 | 0.84857646 | 9.44E-15 |
| | ISG15 | 0.82724423 | 5.18E-17 |
| | FEV | 0.81770723 | 3.27E-09 |
| | GUCY2C | 0.80399178 | 4.64E-14 |
| | RAMP1 | 0.80390259 | 2.45E-13 |
| | C15orf48 | 0.79627586 | 2.56E-30 |
| | HSPA1A | 0.79213823 | 6.01E-11 |
| | CXCL12 | 0.77664092 | 4.10E-16 |
| | PAM | 0.76879532 | 3.18E-19 |
| | SEZ6L | 0.75968371 | 4.28E-27 |
| | FXYD2 | 0.75562301 | 0.00166728 |
| | CHGA | 0.7494752 | 1.63E-25 |
| | ASCL1 | 0.69631919 | 0.00012541 |
| | MAFB | 0.68021658 | 6.96E-14 |
| | RGS9 | 0.68020767 | 2.00E-22 |
| | GCH1 | 0.67314402 | 2.87E-21 |
| | RP11-279F6.1 | 0.65388567 | 6.65E-09 |
| | WFDC2 | 0.65133335 | 2.86E-16 |
| | HSPB1 | 0.64429653 | 2.87E-12 |
| | IFI6 | 0.64402879 | 7.07E-25 |
| | CLDN3 | 0.63748219 | 8.09E-21 |
| | AC009014.3 | 0.61817642 | 1.89E-16 |
| | C12orf75 | 0.61341069 | 8.35E-14 |
| | HLA-B | 0.60411727 | 2.97E-16 |
| | PTPRN | 0.59283695 | 4.94E-23 |
| | LINC00261 | 0.58999555 | 1.71E-09 |
| | ETV1 | 0.58263018 | 3.27E-13 |
| | CYBA | 0.57910221 | 1.21E-17 |
| | FAM46B | 0.57774928 | 1.81E-14 |
| | LY6H | 0.57595111 | 5.92E-10 |
| | HLA-C | 0.5571406 | 1.29E-17 |
| | HES1 | 0.5568618 | 0.01859399 |
| | MT-ND4L | 0.55588667 | 1.66E-14 |
| | SPINK1 | 0.54124436 | 2.16E-07 |
| | MDK | 0.54081909 | 1.21E-06 |
| | PPY | 0.53417065 | 1.66E-21 |
| | RAB3B | 0.53256072 | 1.68E-11 |
| | APOE | 0.53055919 | 3.72E-12 |
| | NAV2 | 0.52619448 | 1.44E-14 |
| | SCG2 | 0.51930402 | 1.50E-05 |
| | TMSB4X | 0.51453668 | 4.90E-05 |
| | DSP | 0.50794817 | 2.14E-10 |
| | LCOR | 0.50065824 | 1.24E-19 |
| **Downregulated** | TTR | -2.1840245 | 2.48E-52 |
| | CRYBA2 | -2.0521569 | 4.99E-67 |
| | SLC30A8 | -1.7606669 | 1.60E-42 |
| | MT1G | -1.4277739 | 0.0097721 |
| | NEFM | -1.3136923 | 1.40E-11 |
| | GCG | -1.0470691 | 4.83E-36 |
| | MT1X | -1.0106086 | 0.03612605 |
| | ACVR1C | -1.0016424 | 1.10E-46 |
| | NPTX2 | -0.9677836 | 1.55E-11 |
| | RP11-788H18.1 | -0.9259451 | 3.40E-22 |
| | HADH | -0.8920445 | 3.15E-22 |
| | RTN1 | -0.8858712 | 6.72E-34 |
| | SMC2 | -0.8849714 | 2.72E-16 |
| | ASPH | -0.8531848 | 9.22E-20 |
| | EPHX1 | -0.8363238 | 8.25E-28 |
| | PCDH7 | -0.8161754 | 6.93E-17 |
| | EGFL7 | -0.8028547 | 1.78E-15 |
| | TLE4 | -0.7756815 | 1.48E-14 |
| | HPCA | -0.7358968 | 4.32E-28 |
| | AIF1 | -0.70121 | 1.30E-23 |
| | TUBB2B | -0.6797651 | 2.46E-17 |
| | LBH | -0.676144 | 1.69E-12 |
| | SLC25A5 | -0.6717014 | 6.83E-20 |
| | HMGB3 | -0.6698967 | 1.85E-18 |
| | SNORA76 | -0.6643836 | 2.98E-19 |
| | STMN2 | -0.6572494 | 7.77E-21 |
| | ROBO2 | -0.6440677 | 5.72E-39 |
| | TNFRSF21 | -0.6343054 | 3.32E-17 |
| | HNRNPA1 | -0.6315225 | 2.04E-27 |
| | ALDH1A1 | -0.6303556 | 4.42E-09 |
| | FTL | -0.6107457 | 5.93E-26 |
| | CHODL | -0.6075175 | 6.93E-25 |
| | NME1 | -0.6039177 | 2.97E-21 |
| | ANGPT2 | -0.5926007 | 2.03E-15 |
| | PPP1R1A | -0.5921187 | 3.13E-23 |
| | SURF4 | -0.5745254 | 1.76E-17 |
| | SVIP | -0.5743591 | 3.82E-07 |
| | FGF12 | -0.5677965 | 2.57E-21 |
| | PLXNA2 | -0.5503125 | 2.38E-08 |
| | RAN | -0.5452398 | 4.38E-21 |
| | PCSK2 | -0.5311741 | 1.66E-08 |
| | APOA2 | -0.5283145 | 4.51E-10 |
| | SNHG8 | -0.5262266 | 6.64E-14 |
| | KRT19 | -0.5223486 | 4.34E-09 |
| | MAN1A1 | -0.5214265 | 1.72E-11 |
| | TUBB | -0.5134801 | 1.90E-13 |
| | LINC00643 | -0.5048979 | 2.74E-15 |

**Supplementary Table 8. Differentially Expressed genes from MAFB +/+ and -/- cells in GCG PPY PYY GAST high (Alpha-like) cells at D20 (P_adj<0.1, FC>0.5)**

| | gene | avg_logFC | p_val_adj |
|---|---|---|---|
| **Upregulated** | GAST | 2.34845121 | 4.24E-33 |
| | SST | 1.87687227 | 6.10E-44 |
| | APOC1 | 1.42598852 | 2.08E-34 |
| | PPY | 1.39317369 | 9.42E-10 |
| | GHRL | 1.20480579 | 1 |
| | TPPP3 | 1.19890362 | 3.37E-28 |
| | CALB2 | 1.0297454 | 4.96E-12 |
| | LY6E | 1.02849752 | 9.55E-25 |
| | TIMP3 | 0.97653856 | 6.30E-21 |
| | VIM | 0.94252217 | 2.57E-17 |
| | FXYD2 | 0.90341815 | 7.48E-06 |
| | MAFB | 0.86993285 | 5.57E-33 |
| | PEG10 | 0.83442657 | 0.00310048 |
| | APOE | 0.80874644 | 2.76E-25 |
| | RGS4 | 0.80821309 | 5.77E-24 |
| | ISG15 | 0.76157627 | 3.34E-21 |
| | LYZ | 0.75428174 | 0.01653335 |
| | AMBP | 0.75381568 | 1.68E-13 |
| | HSPA1B | 0.73811796 | 1.77E-22 |
| | C10orf10 | 0.72203225 | 4.91E-07 |
| | NEAT1 | 0.7071935 | 7.69E-06 |
| | PYY | 0.6740438 | 1 |
| | MLF1 | 0.67158036 | 3.98E-19 |
| | FOXJ1 | 0.66121597 | 3.15E-06 |
| | TMOD1 | 0.65144922 | 4.25E-22 |
| | BAMBI | 0.64792289 | 6.78E-17 |
| | HSP90AA1 | 0.63034606 | 3.50E-29 |
| | C1orf192 | 0.59054142 | 8.24E-08 |
| | TDO2 | 0.58867598 | 0.00101285 |
| | PIFO | 0.57029439 | 7.96E-13 |
| | TMX4 | 0.5698137 | 7.19E-11 |
| | HSPA1A | 0.56574268 | 3.53E-10 |
| | DSP | 0.55331361 | 1.13E-15 |
| | RGS9 | 0.55011204 | 3.41E-19 |
| | AGT | 0.54569588 | 1.97E-05 |
| | GC | 0.54338691 | 0.43178532 |
| | MDK | 0.54269379 | 4.40E-12 |
| | SEZ6L | 0.5424836 | 1.97E-19 |
| | RPL17 | 0.53597572 | 6.38E-29 |
| | MYO10 | 0.526973 | 6.49E-16 |
| | TXNDC12 | 0.52233686 | 3.75E-19 |
| | MYH10 | 0.51451642 | 1.90E-17 |
| | UBE2L6 | 0.514254 | 2.58E-18 |
| | C8orf4 | 0.51300744 | 1.17E-07 |
| | HEPACAM2 | 0.51253835 | 5.94E-12 |
| | DNAJB1 | 0.51086168 | 3.59E-15 |
| | EIF4A2 | 0.50786082 | 7.53E-26 |
| | SPTSSB | 0.5016538 | 8.17E-09 |
| | | | |
| **Downregulated** | INS | -2.6998144 | 5.21E-122 |
| | GCG | -2.3085079 | 3.86E-73 |
| | CRYBA2 | -2.0366977 | 2.67E-113 |
| | APOA2 | -1.7674806 | 7.56E-31 |
| | ACVR1C | -1.4741353 | 2.27E-72 |
| | EPHX1 | -1.0894711 | 9.60E-75 |
| | TTR | -1.0717345 | 8.43E-31 |
| | SLC30A8 | -1.0484865 | 3.94E-49 |
| | PPP1R1A | -0.8705498 | 3.16E-51 |
| | HMGB3 | -0.8705292 | 2.63E-31 |
| | ALDH1A1 | -0.8406196 | 3.10E-23 |
| | GAL | -0.8321412 | 3.24E-07 |
| | PCSK2 | -0.8142138 | 4.87E-39 |
| | RP11-788H1: | -0.7751951 | 2.08E-30 |
| | STMN2 | -0.6887093 | 2.25E-24 |
| | AIF1 | -0.6817355 | 3.21E-35 |
| | SLC7A8 | -0.6718028 | 2.57E-43 |
| | SERPINI1 | -0.6313049 | 7.91E-23 |
| | MAN1A1 | -0.5520895 | 2.80E-16 |
| | C2CD4A | -0.5212733 | 4.15E-19 |
| | MT-ATP6 | -0.5107123 | 4.85E-24 |
| | NPTX2 | -0.5058441 | 7.00E-27 |

**Supplementary Table 9. Differentially Expressed genes from MAFB +/+ and -/- cells in SST HHEX high (Delta-like) cells at D20 (P_adj<0.1, FC>0.5)**

| | Gene | avg_logFC | p_val_adj |
|---|---|---|---|
| **Upregulated** | PPY | 1.16921263 | 0.00065802 |
| | TPPP3 | 0.94230623 | 4.95E-05 |
| | SST | 0.83167127 | 3.23E-07 |
| | APOC1 | 0.7776412 | 0.00014862 |
| | MAFB | 0.71361123 | 2.97E-07 |
| | APOE | 0.70040977 | 1.17E-05 |
| | LY6E | 0.6953392 | 0.00016574 |
| | ISG15 | 0.69312906 | 2.74E-05 |
| | PYY | 0.6342115 | 0.00345824 |
| | C1orf192 | 0.56016347 | 0.01327972 |
| | IFI6 | 0.55576006 | 1.14E-11 |
| | | | |
| **Downregulated** | INS | -2.8150699 | 3.58E-59 |
| | CRYBA2 | -2.2224018 | 4.01E-55 |
| | ACVR1C | -1.3812356 | 3.03E-35 |
| | GCG | -1.143319 | 4.24E-46 |
| | TTR | -1.1143138 | 5.29E-20 |
| | EPHX1 | -1.0512858 | 5.57E-33 |
| | PPP1R1A | -1.024554 | 1.87E-36 |
| | MT1X | -0.9083369 | 0.00957171 |
| | HADH | -0.8972487 | 2.75E-21 |
| | STMN2 | -0.8549379 | 5.11E-12 |
| | MAN1A1 | -0.8211267 | 3.64E-19 |
| | NEUROD1 | -0.8039444 | 1.83E-12 |
| | FABP5 | -0.7611367 | 1.93E-13 |
| | IGFBP5 | -0.7404403 | 7.73E-05 |
| | KRT19 | -0.7397835 | 2.47E-09 |
| | HMGB3 | -0.7186303 | 1.31E-11 |
| | ERO1LB | -0.7150173 | 1.94E-09 |
| | C9orf16 | -0.6663357 | 7.60E-09 |
| | MT-ATP6 | -0.6039107 | 7.24E-15 |
| | PCSK2 | -0.5968298 | 1.43E-09 |
| | SLC30A8 | -0.5885128 | 4.54E-05 |
| | PLCE1 | -0.5561556 | 2.75E-11 |
| | FTL | -0.5540723 | 2.65E-10 |
| | AIF1 | -0.5484026 | 4.55E-08 |
| | MT-ND2 | -0.5467455 | 3.56E-13 |
| | TUBB2B | -0.5396288 | 1.38E-05 |
| | RPS21 | -0.5078175 | 6.78E-12 |
| | SNORA76 | -0.5072658 | 2.52E-07 |
| | IGFBP2 | -0.5026126 | 0.0011693 |
| | SLC7A8 | -0.5016193 | 6.41E-07 |

**Supplementary Table 10. hESC differentiation into α-like cells – Media components**

| Day | Media | Supplement |
|---|---|---|
| D0 | Seed hESCs 5.5 million cells/ well in 6 well plates with D0 media | |
| | Day 0 | HERβ1 10ng/ml; Activin A 10ng/ml; FGF-2 4.16ng/ml |
| D1 | Rinse cells with DPBS (w/o $Ca^{2+}$, $Mg^{2+}$) for 5 min and added Day 1 differentiation media | |
| | Day 1 | FBS 0.2%; Activin A 100ng/ml; ITS 0.02X; Wnt-3a 50ng/ml |
| D2 | Day 2 | FBS 0.2%; Activin A 100ng/ml; ITS 0.05X |
| D3 | Day 3 | FBS 0.2%; KGF 25ng/ml; ITS 0.1X; TGF-β IV 2.5μM |
| D4-5 | Day 4-5 | FBS 0.2%; KGF 25ng/ml; ITS 0.1X |
| D6-8 | Day 6-8 | Noggin 100 ng/ml; TTNPB 3nM |
| D9-11 | Day 9-11 | Noggin 50 ng/ml; Alk5i II 1μM; DAPT 1μM |
| D12-18 | Day12-18 | Alk5i II 1μM |
| D19-onward | Day 19 | Day 19 media on ward until ~ day 55 |

**Supplementary Table 11. hESC differentiation into α-like cells – Backbone Media Formulation**

| Day | Media components |
|---|---|
| D0 | DMEM/F-12 1xGlutamax 1xMEM-NEAA 1x 2-Mercaptoethanol 4% KSR |
| D1-5 | RPMI 1xGlutamax |
| D6-8 | DMEM 1xGlutamax |
| D9-11 | DMEM/F-12 1xGlutamax 1xMEM-NEAA 1xB27 |
| D11-54 | DMEM/F-12 1xGlutamax 1xMEM_NEAA 1xB27 0.2%BSA |

**Supplementary Table 12. Chemicals and media components**

| Components | Vendor | Cat. No. |
|---|---|---|
| DMEM | Life Tech | 11960-051 |
| DMEM-F12 | Life Tech | 11320-033 |
| RPMI | Mediatech | 15-040-CM |
| HERβ1(Heregulin-β1) | PeproTech | 100-03 |
| Activin A | R&D | 338-AC/CF |
| FGF-2 | R&D | 233-FB/CF |
| FBS | Corning | 35-011-CV |
| ITS (Insulin-Transferrin-Selenium) | Gibco | 41400045 |
| Wnt-3a | R&D | 5036-WN-010/CF |
| TGF-β IV (TGF-β RI Kinase Inhibitor IV) | CalBiochem | 616454-2MG |
| KGF (FGF-7) | R&D | 251-KG-050/CF |
| Noggin | R&D | 3344-NG |
| TTNPB | Sigma-Aldrich | T3757-10MG |
| Alk5i II (ALK5 Inhibitor 2) | Axxora | ALX-270-445-M001 |
| DAPT | Sigma-Aldrich | D5942-5MG |
| Glutamax | Gibco | 35050-061 |
| MEM-NEAA | Gibco | 11140-050 |
| 2-Mercaptoethanol | Emd-Millipore | ES-007-E |
| KSR (KnockOut™ Serum replacement) | Gibco | 10828-028 |
| B27 (NeuroCult™ SM1 Neuronal) | Stemcell Technologies | 5711 |
| BSA | Sigma-Aldrich | A4503-50G |
| Doxycycline | Sigma-Aldrich | D9891 |
| LDN | Fisher | 04-0074-10 |

**Supplementary Table 13. CRISPR gRNA target sequences for targeting AAVS1 locus**

| Primer | Sequence (5'to 3') |
| --- | --- |
| AAVS1-cr1-ex | GATGACCGAGTACAAGCCCA |
| AAVS1-cr2-ex | GACAGTACTAAGCTTTACTA |

**Supplementary Table 14. Antibodies used for FACS analysis**

| Manufacturer | Antibody | Catalogue No. | Dilution | Fluorophore |
|---|---|---|---|---|
| BD Pharmingen/Fisher | Foxa2 | 561589 | 1:100 | PE |
| BD Pharmingen/Fisher | Sox17 | 562205 | 1:100 | Alexa F. 488 |
| Biolegend | TRA-1-60-R | 330605 | 1:200 | Alexa F. 647 |
| BD Pharmingen/Fisher | PDX1 | 562161 | 1:50 | PE |
| BD Pharmingen/Fisher | NKX6.1 | 563338 | 1:50 | Alexa F. 647 |
| BD Pharmingen/Fisher | C-peptide | 051109MI | 1:200 | Alexa F. 488 |
| Fisher | PAX6 | 562249 | 1:50 | Alexa F. 647 |
| BD Pharmingen/Fisher | NKX2.2 | 564730 | 1:50 | PE |
| BD Pharmingen/Fisher | Chromogranin A | BDB564563 | 1:50 | PE |
| Sigma | Glucagon | G2654 | 1:1000 | Alexa F. 647 |
| BD Pharmingen/Fisher | KI67 | 550609 | 1:20 | Alexa F. 647 |
| Millipore | ARX | MABN102 | 1:200 | PE |
| DAKO | SST | A0566 | 1:250 | Alexa F. 568 |
| Peninsula Laboratories International, Inc. | PPY | T-4088 | 1:500 | Alexa F. 647 |

**Supplementary Table 15. Antibodies used for IF analysis**

| Antibody | Species | IF Dilution | Company | Catalogue No. |
|---|---|---|---|---|
| Somatostatin | Rabbit | 1:250 | DAKO | A0566 |
| Somatostatin | Sheep | 1:250 | Biogenesis | 8330-0350 |
| PDX1 | Goat | 1:100 | R&D | AF2419 |
| NKX6.1 | Mouse | 1:100 | Hybridoma Bank, U Iowa | F55A10-c |
| Sox17 | Goat | 1:200 | R&D | AF1924 |
| FoxA2 | Rabbit | 1:1000 | Millipore | 07-633 |
| KI-67 | Mouse | 1:400 | BD | 550609 |
| NGN3 | Sheep | 1:200 | R&D | AF3444 |
| Glucagon | Rabbit | 1:200 | Linco | 4030-01F |
| Glucagon | Mouse | 1:250 | Sigma | G2654 |
| C-peptide | Rat | 1:1000 | DSHB | GN-1D4-C |
| NGN3 | Sheep | 1:200 | R&D | AF3444 |
| ARX | Mouse | 1:200 | Millipore | MABN102 |
| HHEX | Rabbit | 1:200 | R&D | MAB83771-100 |
| PPY | Rabbit | 1:2000 | Peninsula Laboratories International, Inc. | T-4088 |
| MAFB | Rabbit | 1:250 | Sigma | HPA005653 |

**Supplementary Table 16. Antibodies used for WB analysis**

| Antibody | Species | IF Dilution | Company | Catalogue No. |
|---|---|---|---|---|
| GAPDH | Mouse | 1:3000 | Santa Cruz | sc-32233 |
| MAFB | Rabbit | 1:300 | Sigma | HPA0055653 |
| Somatostatin | Rabbit | 1:2000 | Dako | A0566 |
| Vinculin | Mouse | 1:10,000 | Santa Cruz | sc-73614 |

**Supplementary Table 17. qPCR primer sequences**

| Primer Name | Forward Sequence | Reverse Sequence |
|---|---|---|
| ALDH1A3 | TCTGAGGGTTCTAATACAGCCC | CCCTGGAGACGATGGATACAG |
| ARX | GTGCAAGGCTCCCCTAAGAG | CGTTCTCGCGGTACGACTT |
| CHGA | TGACCTCAACGATGCATTTC | CTGTCCTGGCTCTTCTGCTC |
| GAPDH | CTCACCGGATGCACCAATGTT | CGCGTTGCTCACAATGTTCAT |
| GCG | GAGATTTCCCAGAAGAGGTCG | TGGCGGCAAGATTATCAAGAA |
| GHRL | CAACGCCCCCTTTGATGTTG | CTGCTGGTACTGAACCCCTG |
| HHEX | GCGAGAGACAGGTCAAAACC | AGGGCGAACATTGAGAGCTA |
| HNF6 | ATGTCCAGCGTCGAACTCTAC | TGCTTTGGTACAAGTGCTTGAT |
| INS | AGAGGCCATCAAGCAGATCACTGT | ACAGGTGTTGGTTCACAAAGGCTG |
| MAFA | GAGAGCGAGAAGTGCCAACT | TTCTCCTTGTACAGGTCCCG |
| MAFB | CATAGAGAACGTGGCAGCAA | ATGCCCGGAACTTTTTCTTT |
| NEUROD1 | ATGACCAAATCGTACAGCGAG | GTTCATGGCTTCGAGGTCGT |
| NGN3 | TAAGAGCGAGTTGGCACTGAGCAA | TTTGAGTCAGCGCCCAGATGTAGT |
| NKX2.2 | GGAGCTTGAGTCCTGAGGG | TCTACGACAGCAGCGACAAC |
| NKX6.1 | CCGAGTCCTGCTTCTTCTTG | ATTCGTTGGGGATGACAGAG |
| PAX4 | CTACCGCACAGGTGTCTTGG | CGTTGGATTTCCCAGGCAA |
| PAX6 | TGGGCAGGTATTACGAGACTG | ACTCCCGCTTATACTGGGCTA |
| PDX1 | TACTGGATTGGCGTTGTTTGTGGC | AGGGAGCCTTCCAATGTGTATGGT |
| PPY | AGTGTACCCAGGGGACAATGC | CAGCATGTTGAGTGTATCTACGGA |
| Pre-INS | GTGAACCAACACCTGTGCGG | AGGGGCAGCAATGGGCAGTT |
| PTF1A | TTCACCGACCAGTCTTCACG | GTGGCTAAGGAACTCCACCT |
| SOX9 | AGCGAACGCACATCAAGAC | CTGTAGGCGATCTGTTGGGG |
| SST | CGCTGTCCATGGTCCTG | GGGCATCATTCTCCGTCT |
