# Supplementary material for: Loss of the transcription factor MAFB limits β-cell derivation from human PSCs
Source: Nat Commun. 2020 Jun 2;11:2742. doi: 10.1038/s41467-020-16550-9 (PMC7265500; doi:10.1038/s41467-020-16550-9)
Supplement: Supplementary file 3 — Reporting Summary [file 41467_2020_16550_MOESM3_ESM.pdf]

nature research

Corresponding author(s): Matthias Hebrok

Last updated by author(s): Apr 13, 2020

Reporting Summary

Nature Research wishes to improve the reproducibility of the work that we publish. This form provides structure for consistency and transparency in reporting. For further information on Nature Research policies, see Authors & Referees and the Editorial Policy Checklist .

Statistics

For all statistical analyses, confirm that the following items are present in the figure legend, table legend, main text, or Methods section.

n/a	Confirmed	
☐	☒	The exact sample size (*n*) for each experimental group/condition, given as a discrete number and unit of measurement
☐	☒	A statement on whether measurements were taken from distinct samples or whether the same sample was measured repeatedly
☐	☒	The statistical test(s) used AND whether they are one- or two-sided<br>*Only common tests should be described solely by name; describe more complex techniques in the Methods section.*
☐	☒	A description of all covariates tested
☐	☒	A description of any assumptions or corrections, such as tests of normality and adjustment for multiple comparisons
☐	☒	A full description of the statistical parameters including central tendency (e.g. means) or other basic estimates (e.g. regression coefficient) AND variation (e.g. standard deviation) or associated estimates of uncertainty (e.g. confidence intervals)
☐	☒	For null hypothesis testing, the test statistic (e.g. *F*, *t*, *r*) with confidence intervals, effect sizes, degrees of freedom and *P* value noted<br>*Give P values as exact values whenever suitable.*
☒	☐	For Bayesian analysis, information on the choice of priors and Markov chain Monte Carlo settings
☒	☐	For hierarchical and complex designs, identification of the appropriate level for tests and full reporting of outcomes
☒	☐	Estimates of effect sizes (e.g. Cohen's *d*, Pearson's *r*), indicating how they were calculated

Our web collection on statistics for biologists contains articles on many of the points above.

Software and code

Policy information about availability of computer code

Data collection	BD FACSDiva v. 6.0 software was used for flow cytometry data collection using BD LSRII.
Data analysis	Graphad Prism7/8<br>FlowJo v10<br>Microsoft Excel v.16.35<br>Cell Ranger software v2.1.1<br>STAR aligner v.2.7<br>R package Seurat v.2.3.4<br>ImageJ v1.51<br>DESeq2 v.1.16.0<br>TrimGalore 0.4.0<br>MACS2.1.0<br>Bowtie2 v. 2.3.5<br>MAST v. 3.10<br>FastQC 0.11.7<br>No custom code was used.

For manuscripts utilizing custom algorithms or software that are central to the research but not yet described in published literature, software must be made available to editors/reviewers. We strongly encourage code deposition in a community repository (e.g. GitHub). See the Nature Research guidelines for submitting code & software for further information.

Data

Policy information about availability of data

All manuscripts must include a data availability statement. This statement should provide the following information, where applicable:

- Accession codes, unique identifiers, or web links for publicly available datasets
- A list of figures that have associated raw data
- A description of any restrictions on data availability

RNA sequences for the single-cell RNA-sequencing analyses reported in this paper have been deposited in the GEO database with the accession code GSE145347 [https://www.ncbi.nlm.nih.gov/geo/query/acc.cgi?acc=GSE145347]. MAFB ChIP seq data from EndoC-βH2 cells was uploaded to ArrayExpress, accession E-MTAB-8612 [https://www.ebi.ac.uk/arrayexpress/experiments/E-MTAB-8612/]. MAFB ChIP seq data from human islets are available at Islet Regulome 80 [http://www.isletregulome.org/] and can be viewed using the UCSC Genome browser 81 [https://genome.ucsc.edu/cgi-bin/hgTracks?db=hg19&lastVirtModeType=default&lastVirtModeExtraState=&virtModeType=default&virtMode=0&nonVirtPosition=&position=chr1%3A1%2D2&hgsid=815997045_p41DJpFchqeSccV95h3OjaUelETa]. The authors declare that all data supporting the findings Browser, of this study are available within the article and its supplementary information files or from the corresponding author (MH) upon reasonable request.

Figures with associated raw data:
Figure 4A,B
Figure S5A,B,C
Figure S6A,B,C,D
Figure S7A
Figure S8

Field-specific reporting

Please select the one below that is the best fit for your research. If you are not sure, read the appropriate sections before making your selection.

[x] Life sciences   [ ] Behavioural & social sciences   [ ] Ecological, evolutionary & environmental sciences

For a reference copy of the document with all sections, see nature.com/documents/nr-reporting-summary-flat.pdf

Life sciences study design

All studies must disclose on these points even when the disclosure is negative.

Sample size	Statistical methods were not used to determine sample size. Sample sizes were chosen based on availability of experimental samples. These sample sizes are sufficient since the study is very thorough and we have used a variety of experimental techniques to confirm the findings
Data exclusions	Stem Cell differentiations that did not pass a minimum criteria of 85% SOX17+/FOXA2+ double positive cells at the early stage of defintive endoderm were excluded from further analysis. This criteria was preselected because the formation of definitive endoderm is the first step toward pancreatic differentiation. When cells don't pass the above criteria, the spheres do not form pancreatic endoderm efficiently and start falling apart on further culture.
Replication	At least 3 or more biological replicates were considered for the analysis using stage-matched controls as a reference.<br>Using the above criteria, all replication attempts were successful.<br>Additionally, we used a second hPSC cell line to confirm the major findings of the study.<br>Samples of different stages of differentiation sent to other researchers confirmed our findings.
Randomization	There was no randomization applicable for the in vitro experiments as cell lines had to be identified to establish independent clones . For transplantation experiments in vivo, mice were randomly assigned to receive either MAFB+/+ or MAFB-/- cells.
Blinding	Investigators were blinded to samples collected from in vivo transplantation studies.<br>Investigators were not blinded to group allocation as phenotypes were identifiable during the analysis.

Reporting for specific materials, systems and methods

We require information from authors about some types of materials, experimental systems and methods used in many studies. Here, indicate whether each material, system or method listed is relevant to your study. If you are not sure if a list item applies to your research, read the appropriate section before selecting a response.

Materials & experimental systems

n/a	Involved in the study
☐	☒ Antibodies
☐	☒ Eukaryotic cell lines
☒	☐ Palaeontology
☐	☒ Animals and other organisms
☒	☐ Human research participants
☒	☐ Clinical data

Methods

n/a	Involved in the study
☐	☒ ChIP-seq
☐	☒ Flow cytometry
☒	☐ MRI-based neuroimaging

Antibodies

Antibodies used

Somatostatin Rabbit 1:250 DAKO A0566
Somatostatin Sheep 1:250 Biogenesis 8330-0350
PDX1 Goat 1:100 R&D AF2419
NKX6.1 Mouse 1:100 Hybridoma Bank, U Iowa F55A10-c
Sox17 Goat 1:200 R&D AF1924
FoxA2 Rabbit 1:1000 Millipore 07-633
KI-67 Mouse 1:400 BD 550609
NGN3 Sheep 1:200 R&D AF3444
Glucagon Rabbit 1:200 Linco 4030-01F
Glucagon Mouse 1:250 Sigma G2654
C-peptide Rat 1:1000 DSHB GN-1D4-C
NGN3 Sheep 1:200 R&D AF3444
ARX Mouse 1:200 Millipore MABN102
HHEX Rabbit 1:200 R&D MAB83771-100
PPY Rabbit 1:2000 Peninsula Laboratories International, Inc. T-4088
MAFB Rabbit 1:250 Sigma HPA005653
GAPDH Mouse 1:3000 Santa Cruz sc-32233
Vinculin Mouse 1:10,000 Santa Cruz sc-73614
BD Pharmingen/Fisher Foxa2 561589 1:100 PE
BD Pharmingen/Fisher Sox17 562205 1:100 Alexa F. 488
Biolegend TRA-1-60-R 330605 1:200 Alexa F. 647
BD Pharmingen/Fisher PDX1 562161 1:50 PE
BD Pharmingen/Fisher NKX6.1 563338 1:50 Alexa F. 647
BD Pharmingen/Fisher C-peptide 051109MI 1:200 Alexa F. 488
Fisher PAX6 562249 1:50 Alexa F. 647
BD Pharmingen/Fisher NKX2.2 564730 1:50 PE
BD Pharmingen/Fisher Chromogranin A BDB564563 1:50 PE
Sigma Glucagon G2654 1:1000 Alexa F. 647
BD Pharmingen/Fisher KI67 550609 1:20 Alexa F. 647
Millipore ARX MABN102 1:200 PE
DAKO SST A0566 1:250 Alexa F. 568
Peninsula Laboratories International, Inc. PPY T-4088 1:500 Alexa F. 647
MafB Recombinant Monoclonal Antibody [BLR046F], A700-046
Goat anti-SST, Santa Cruz (D-20) sc-7819

Validation

The primary antibodies for immunofluorescent staining were validated on Human pancreatic sections using various dilutions (according to the manufacturer's instructions). Primary antibody only- and secondary antibody only- controls were used to evaluate the specificity of the antibodies to the respective epitopes.
The expression patterns observed with all the antibodies included in our paper display stage-specific expression by immunostaining and flow cytometry.

Eukaryotic cell lines

Policy information about cell lines

Cell line source(s)

The human ES-cell line, Mel1 INSGFP/W used in the study is available from Prof. Ed Stanley at Murdoch Children's Research Institute under MTA in place with Murdoch Children's Research Institute.
hIPSCs are comercially available from Gibco #A18945.

Authentication

None of the cell lines were authenticated.

Mycoplasma contamination

All cell lines tested negative for mycoplasma contamination.

Commonly misidentified lines (See ICLAC register)	No cell lines used in this study were reported in the ICLAC register.

Animals and other organisms

Policy information about studies involving animals; ARRIVE guidelines recommended for reporting animal research

Laboratory animals	NOD.Cg-Prkdcscid Il2rgtm1Wjl/SzJ (NSG) male and female mice at 8-12 weeks old were obtained from Jackson Laboratories. Mice were maintained at a constant humidity between 30-70% and temperature 68-79 degrees Fahrenheit, under a 12-h light/dark cycle and had free access to food and water until experiment initiation.
Wild animals	No wild animals we used in this study.
Field-collected samples	Field samples were not collected for this study.
Ethics oversight	All animal experiments were performed in accordance with the International Animal Care and Use Committee (IACUC) regulations at UCSF.

Note that full information on the approval of the study protocol must also be provided in the manuscript.

ChIP-seq

Data deposition

- [x] Confirm that both raw and final processed data have been deposited in a public database such as GEO.
- [x] Confirm that you have deposited or provided access to graph files (e.g. BED files) for the called peaks.

Data access links<br>*May remain private before publication.*	E-MTAB-8612<br>http://www.isletregulome.org
Files in database submission	5359-RS-0001_trimmed.fq.gz, 5359-RS-0003_trimmed.fq.gz, 5359-RS-0005_trimmed.fq.gz
Genome browser session (e.g. UCSC)	E-MTAB-8612<br>http://www.isletregulome.org

Methodology

Replicates	Three treatment replicates were collected
Sequencing depth	20-24M/sample
Antibodies	anti-MAFB (Bethyl BL658)
Peak calling parameters	Read mapping was performed on TrimGalore-trimmed FastQ files via Bowtie2, using bowtie2_ucsc_hg19 genome reference. For each treatment mapped file (sorted BAM), MACS2 was used for peak detection using default parameters and -bw 300.
Data quality	2573 peaks at at FDR 5% and above 5-fold enrichment
Software	Library construction was performed by HudsonAlpha, using HiSeq v4 chemistry and single-ended sequencing was performed on an Illumina HiSeq2500. The 50bp reads from each sample were initially assessed FastQC 0.11.7 76. Following quality-filtering and adapter trimming with TrimGalore 0.4.0 77, the remaining reads were aligned to the UCSC reference human genome (hg19, GRCh37) with Bowtie2 and then further filtered prior to peak calling, which was performed with MACS2.1.0 78. No custom code was used.

Flow Cytometry

Plots

Confirm that:

- [x] The axis labels state the marker and fluorochrome used (e.g. CD4-FITC).
- [x] The axis scales are clearly visible. Include numbers along axes only for bottom left plot of group (a 'group' is an analysis of identical markers).
- [x] All plots are contour plots with outliers or pseudocolor plots.
- [x] A numerical value for number of cells or percentage (with statistics) is provided.

Methodology

Sample preparation	Cells were dissociated using accumax. Cells were then fixed with 4% PFA.

Instrument	LSRFortessa X20 Dual, Aria II
Software	FACS DIVA / FlowJo software
Cell population abundance	Flow cytometry was used for marker expression analysis of fixed samples. No cell sorting was performed.
Gating strategy	Gating was based on stage matched samples that were unstained or negatively stained for the associated markers and an example of the strategy is shown in Supplementary Figure 16 E.

☒ Tick this box to confirm that a figure exemplifying the gating strategy is provided in the Supplementary Information.
